# Mendelian gene identification through mouse embryo viability screening

**DOI:** 10.1186/s13073-022-01118-7

**Published:** 2022-10-13

**Authors:** Pilar Cacheiro, Carl Henrik Westerberg, Jesse Mager, Mary E. Dickinson, Lauryl M. J. Nutter, Violeta Muñoz-Fuentes, Chih-Wei Hsu, Ignatia B. Van den Veyver, Ann M. Flenniken, Colin McKerlie, Stephen A. Murray, Lydia Teboul, Jason D. Heaney, K. C. Kent Lloyd, Louise Lanoue, Robert E. Braun, Jacqueline K. White, Amie K. Creighton, Valerie Laurin, Ruolin Guo, Dawei Qu, Sara Wells, James Cleak, Rosie Bunton-Stasyshyn, Michelle Stewart, Jackie Harrisson, Jeremy Mason, Hamed Haseli Mashhadi, Helen Parkinson, Ann-Marie Mallon, International Mouse Phenotyping Consortium, John R. Seavitt, Angelina Gaspero, Uche Akoma, Audrey Christiansen, Sowmya Kalaga, Lance C. Keith, Melissa L. McElwee, Leeyean Wong, Tara Rasmussen, Uma Ramamurthy, Kiran Rajaya, Panitee Charoenrattanaruk, Qing Fan-Lan, Lauri G. Lintott, Ozge Danisment, Patricia Castellanos-Penton, Daniel Archer, Sara Johnson, Zsombor Szoke-Kovacs, Kevin A. Peterson, Leslie O. Goodwin, Ian C. Welsh, Kristina J. Palmer, Alana Luzzio, Cynthia Carpenter, Coleen Kane, Jack Marcucci, Matthew McKay, Crystal Burke, Audrie Seluke, Rachel Urban, Genomics England Research Consortium, John C. Ambrose, Prabhu Arumugam, Roel Bevers, Marta Bleda, Freya Boardman-Pretty, Christopher R. Boustred, Helen Brittain, Matthew A. Brown, Mark J. Caulfield, Georgia C. Chan, Greg Elgar, Adam Giess, John N. Griffin, Angela Hamblin, Shirley Henderson, Tim J. P. Hubbard, Rob Jackson, Louise J. Jones, Dalia Kasperaviciute, Melis Kayikci, Athanasios Kousathanas, Lea Lahnstein, Sarah E. A. Leigh, Ivonne U. S. Leong, Javier F. Lopez, Fiona Maleady-Crowe, Meriel McEntagart, Federico Minneci, Jonathan Mitchell, Loukas Moutsianas, Michael Mueller, Nirupa Murugaesu, Anna C. Need, Peter O’Donovan, Chris A. Odhams, Christine Patch, Mariana Buongermino Pereira, Daniel Perez-Gil, John Pullinger, Tahrima Rahim, Augusto Rendon, Tim Rogers, Kevin Savage, Kushmita Sawant, Richard H. Scott, Afshan Siddiq, Alexander Sieghart, Samuel C. Smith, Alona Sosinsky, Alexander Stuckey, Mélanie Tanguy, Ana Lisa Taylor Tavares, Ellen R. A. Thomas, Simon R. Thompson, Arianna Tucci, Matthew J. Welland, Eleanor Williams, Katarzyna Witkowska, Suzanne M. Wood, Magdalena Zarowiecki, Damian Smedley

**Affiliations:** 1 William Harvey Research Institute, Queen Mary University of London, London, UK; 2 MRC Harwell Institute, Harwell, Oxfordshire, UK; 3 Department of Veterinary and Animal Sciences, University of Massachusetts, Amherst, MA USA; 4 Department of Molecular Physiology and Biophysics, Baylor College of Medicine, Houston, TX USA; 5 Department of Molecular and Human Genetics, Baylor College of Medicine, Houston, TX USA; 6 The Hospital for Sick Children, The Centre for Phenogenomics, Toronto, Canada; 7 European Molecular Biology Laboratory-European Bioinformatics Institute, Hinxton, UK; 8 Department of Education, Innovation and Technology, Baylor College of Medicine, Houston, TX USA; 9 Department of Obstetrics and Gynecology, Baylor College of Medicine, Houston, TX USA; 10 Lunenfeld-Tanenbaum Research Institute, Mount Sinai Hospital, The Centre for Phenogenomics, Toronto, Canada; 11 The Jackson Laboratory, Bar Harbor, ME USA; 12 The Mary Lyon Centre, MRC Harwell Institute, Harwell, Oxfordshire, UK; 13 Mouse Biology Program, University of California Davis, Davis, CA USA

## Abstract

**Background:**

The diagnostic rate of Mendelian disorders in sequencing studies continues to increase, along with the pace of novel disease gene discovery. However, variant interpretation in novel genes not currently associated with disease is particularly challenging and strategies combining gene functional evidence with approaches that evaluate the phenotypic similarities between patients and model organisms have proven successful. A full spectrum of intolerance to loss-of-function variation has been previously described, providing evidence that gene essentiality should not be considered as a simple and fixed binary property.

**Methods:**

Here we further dissected this spectrum by assessing the embryonic stage at which homozygous loss-of-function results in lethality in mice from the International Mouse Phenotyping Consortium, classifying the set of lethal genes into one of three windows of lethality: early, mid, or late gestation lethal. We studied the correlation between these windows of lethality and various gene features including expression across development, paralogy and constraint metrics together with human disease phenotypes. We explored a gene similarity approach for novel gene discovery and investigated unsolved cases from the 100,000 Genomes Project.

**Results:**

We found that genes in the early gestation lethal category have distinct characteristics and are enriched for genes linked with recessive forms of inherited metabolic disease. We identified several genes sharing multiple features with known biallelic forms of inborn errors of the metabolism and found signs of enrichment of biallelic predicted pathogenic variants among early gestation lethal genes in patients recruited under this disease category. We highlight two novel gene candidates with phenotypic overlap between the patients and the mouse knockouts.

**Conclusions:**

Information on the developmental period at which embryonic lethality occurs in the knockout mouse may be used for novel disease gene discovery that helps to prioritise variants in unsolved rare disease cases.

**Supplementary Information:**

The online version contains supplementary material available at 10.1186/s13073-022-01118-7.

## Background

The rate of molecular diagnosis through genomics approaches continues to improve. However, the diagnostic yield for Mendelian disorders varies significantly, ranging from 25 to 58% [1, 2] depending on the age of the proband, the type of disorder, the criteria for patient inclusion (e.g. absence of a clear clinical diagnosis, previous attempts to provide a molecular diagnosis) and the availability of sequence data from family members, e.g. familial versus sporadic cases. Despite this progress, a considerable proportion of patients remain without a diagnosis. Potential strategies to address the challenge of undiagnosed patients and advance our understanding of the molecular basis of these disorders include but are not limited to (i) identifying novel Mendelian disease genes [3]; (ii) developing experimental and computational approaches to assess the pathogenicity of variants of unknown significance in known disease genes; (iii) considering expansion of the phenotype of known disease genes [4]; (iv) investigating noncoding, regulatory variants; (v) assessing the contribution of structural variation [5]; (vi) investigating somatic mosaicism; and (vii) exploring alternative modes of inheritance, i.e. digenic or multigenic [2].

With regard to the first approach, the number of genes currently known to be associated with rare disorders comprises 20–25% of the protein coding genome according to OMIM [6]. There are between 200 and 300 new disease-gene associations published every year [7], with many more to be uncovered. Frameworks such as the Clinical Genome Resource or the Genomics England (GEL) PanelApp, a publicly available knowledgebase containing expert curated gene panels related to human disorders, are key to summarise and assess all curated evidence and provide clinical validation for these gene-disease pairs [8, 9]. The number of additional disease-associated genes yet to be identified is estimated to be high, up to 1.5–3 times the number of currently known causative genes of Mendelian conditions [10].

The main approach to identify genes underlying autosomal recessive (AR) disorders has been homozygosity mapping combined with mutation screening in large consanguineous pedigrees. However, this is infrequent in outbred populations, where recessive disorders likely remain underdiagnosed [11]. The use of large exome and sequence datasets, including information on variant frequency and gene intolerance to variation metrics, has been widely implemented in rare disease diagnostic pipelines. Conversely, in large cohorts such as those from UK Biobank [12] and gnomAD [13], we are unlikely to find homozygous loss-of-function (LoF) variants, i.e. complete knockouts, for many genes [14]. A recent study in the European population estimated that every individual is a carrier of at least 2 pathogenic variants in genes known to be associated with AR disease and consequently up to 1% of couples within this population would be at risk of having a child affected by these disorders. This risk increases for consanguineous couples and skeletal disorders and intellectual disabilities [15]. Additionally, variants associated with AR disorders could result in attenuated phenotypes in heterozygous carriers [16]. Hence, identifying biallelic pathogenic variants in rare disease cohorts like the 100,000 Genomes Project (100KGP) [17] remains a crucial task that requires alternative approaches, including evaluating genes not yet associated with disease.

Combining different sources of information can boost the evidence for new disease-gene associations. Integrating research and clinical datasets has proven to be effective at discovering the molecular basis for genetic disorders [18, 19]. Model organism information on viability and cross-species phenotype comparisons in combination with clinical data constitutes another powerful strategy. Some examples include the automatic detection of mouse models for human disease and phenotype-based variant prioritisation using algorithms such as PhenoDigm and Exomiser [20–22]. Additionally, mouse data on essentiality can be used as a discovery and prioritisation tool [23, 24]. We previously developed a gene prioritisation strategy focused on neurodevelopmental disorders by integrating evidence of intolerance to LoF variation from multiple resources and data from large scale sequencing programmes [25]. Through this approach, combining viability data from mice and human cell line screens, we were able to identify a set of developmentally lethal genes, i.e. genes not essential for cell proliferation but required for organism development, which were enriched for autosomal dominant (AD), developmental disease-associated genes. Investigation of clinical cases with de novo variants in developmental lethal genes and phenotypic overlap between the knockout mouse and affected individuals led us to prioritise a set of 9 candidate genes. Two of these genes have since been validated [26, 27].

To improve and expand these successful strategies to other types of disorders, here we again leverage evidence from high-throughput mouse phenotype screens conducted by the International Mouse Phenotyping Consortium (IMPC) to further explore the spectrum of intolerance to LoF variation. For genes with null alleles that result in a lethal phenotype in a primary viability screen (i.e. no live homozygous animals identified between 14 days of age and weaning), the IMPC performs a secondary embryo viability screen to determine a ‘window of lethality’ (WoL) by examining the survival of homozygous null mutants at different embryonic developmental time points [24]. In the present study, we further dissected this set of lethal genes in the mouse with the primary aim of investigating how they can inform human disease gene discovery.

First, we explored these WoL and show how they relate to essentiality inferred from human cell proliferation assays, gene expression across development, intolerance to variation metrics and duplication events. Secondly, we investigated these WoL in the context of human Mendelian disease and found that early-gestation lethal genes in the mouse are correlated with AR disease-associated genes, in particular those involved in inherited metabolic disorders, resulting mainly from enzyme deficiencies [28]. Finally, we developed two gene prioritisation strategies to identify novel candidate genes for this type of disorders: one based on gene similarity to biallelic inborn errors of metabolism (BIEM) genes, a broad category of genes that function in metabolism and impact, or are impacted by most cellular processes [29], and the other based on enrichment of biallelic predicted pathogenic variants among unsolved metabolic disorder cases from the 100KGP [17].

## Methods

### Data sources

#### IMPC mouse data

Mouse primary and secondary viability data were obtained from the IMPC resource [30].

Primary viability data: http://ftp.ebi.ac.uk/pub/databases/impc/all-data-releases/release-15.0/results/viability.csv.gz (DR15) [Downloaded 28.09.21].

Phenotype annotations [31, 32]: DR15.0 / DR16.0.

Embryonic viability data: Detailed information on the primary and secondary viability pipelines, including definitions, procedures and protocols, can be found at https://www.mousephenotype.org/impress/index. These include the following: Viability Primary Screen, Viability E9.5 Secondary Screen, Viability E12.5 Secondary Screen, Viability E14.5-E15.5 Secondary Screen, Viability E18.5 Secondary Screen, Homozygote Viability at Weaning Screen. A full description of the WoL is available (File S1 [33]).

#### Entire set of human protein coding genes with the corresponding mouse orthologues

One-to-one human orthologues were obtained from the HUGO Gene Nomenclature Committee (HGNC) resource [34]: http://ftp.ebi.ac.uk/pub/databases/genenames/hgnc/tsv/locus_groups/protein-coding_gene.txt [Downloaded 28.09.21].

All other gene features used in this study correspond to human orthologue gene annotations. Gene symbols, Ensembl and Uniprot identifiers were converted into HGNC unique identifiers. Where there was any ambiguity about gene id mapping, the annotation was discarded.

#### Human cell proliferation scores

CRISPR knockout screens from the Achilles pipeline (release 21Q3) for 902 cell lines and the corresponding cell line information were obtained from the DepMap portal [35]: https://depmap.org/portal/download/all/ (Achilles_gene_effect_CERES.csv) [Downloaded 28.09.21]. Gene effect scores are direct estimates of the effect of a gene knockout on viability. Thus, a more negative CERES score indicates more depletion in the cell line. Average scores per gene were computed. In order to establish a binary threshold to classify genes as cellular essential and non-essential, previous data on cell essentiality, based on 11 cell lines from 3 different studies, was used to compute F1 scores derived from confusion matrices generated when considering different CERES mean scores and the classification from these 3 studies, and mean score cut-offs of −0.40, −0.45, and −0.55 were found to maximise the F1 scores across the different datasets, similar to the −0.45 threshold estimated with information from 485 cell lines [25, 30].

#### Gene expression across development

Human gene expression (RPKM) across development for brain, cerebellum, heart, kidney, liver, ovary and testis was obtained from Cardoso-Moreira et al. [36] https://apps.kaessmannlab.org/evodevoapp/ [Downloaded 10.08.21].

Data on comparison of temporal trajectories between human genes and their orthologues in mouse for brain and cerebellum was obtained from Cardoso-Moreira et al. [37].

#### Intolerance to variation scores

gnomaAD v2.1.1 constraint metrics [13] (LOEUF, pLI and pRec) and DOMINO scores [38]: https://gnomad.broadinstitute.org/downloads#v2constraint; https://wwwfbm.unil.ch/domino/ [Downloaded 10.08.21] SCoNeS [39] and RVIS [40] scores.

#### Gene duplicates

Annotation of paralogues of human genes was obtained from Ensembl Biomart (Ensembl Genes 104) [41] https://www.ensembl.org/biomart/martview/. Only protein coding paralogues with HGNC ids and % amino acid identity ≥20% were considered [Downloaded 10.08.21].

#### Protein-protein interactions

Human protein network data (scored links between proteins) were obtained from STRING [42] https://stringdb.org/cgi/download?sessionId=%24input%3E%7BsessionId%7D&species_text=Homo+sapiens [Downloaded 13.08.21].

#### Pathways

Lowest level pathways were obtained from Reactome [43] https://reactome.org/download/current/UniProt2Reactome.txt and https://reactome.org/download/current/ReactomePathways.txt [Downloaded 10.08.21].

#### Protein families

PFAM protein families [44] were obtained through Ensembl biomart (Ensembl Genes 104) https://www.ensembl.org/biomart/martview/ [Downloaded 10.08.21].

#### Protein complex

Corum protein complex information [45] was accessed at: https://mips.helmholtzmuenchen.de/corum/#download [Downloaded 13.08.21].

### Disease features

#### Mendelian disease genes, disease category and mode of inheritance

Diagnostic grade ‘green’ genes with sufficient evidence for disease association and their corresponding modes of inheritance were obtained from GEL PanelApp, a publicly available knowledge base containing gene panels related to human disorders [9]. A total number of 313 gene panels (excluding additional findings) were investigated. Information on allelic requirement and level of evidence of disease causation was retrieved for our analysis. Genes from 186 gene panels containing level 2 disease category information (21 categories) were used for the analysis based on disease classification https://PanelApp.genomicsengland.co.uk/panels/ [Downloaded 10.08.21].

#### Human Phenotype Ontology annotations

Phenotypes were obtained from the Human Phenotype Ontology (HPO) (genes to phenotypes) [46] and mapped to the top level of the ontology, broadly corresponding to the physiological system affected. Co-occurrence with the most frequent systems affected (neurological and musculoskeletal) were computed for early lethal genes (EL) versus non early lethal genes (NEL). https://hpo.jax.org/app/download/annotation; https://raw.githubusercontent.com/obophenotype/human-phenotype-ontology/master/hp.obo [Downloaded 23.08.21, HPO notes: format-version: 1.2 data-version: hp/releases/2021-08-02].

#### Prenatal and perinatal lethal genes in humans

A set of 624 genes associated to prenatal and perinatal lethality based on OMIM records obtained from Dawes et al. [6, 47] were used for the analysis. OMIM text fields across the database were queried through the API for terms associated with early lethality, before or shortly after birth. A total of 86 search terms were queried, including ‘early death’, ‘fetal death’, ‘lethal AND prenatal’, ’lethal AND perinatal’, ‘lethal AND neonatal’ among others. The clinical descriptions for each of the initial hits were reviewed to exclude genes with no explicit evidence.

### Prediction of early lethal genes

Several genes have undergone the IMPC primary viability assessment, but the embryonic stage at which lethality occurs has not yet been investigated. To increase the pool of potential candidate early lethal genes, we built a classifier using human cell proliferation scores from 902 lines as predictor variables. For that we used the R implementation of Generalized Additive Model Selection, *gamsel* [48]. The training set consisted of 895 genes, 430 early-lethal (EL) and 465 non-early lethal (NEL)*.* Imputation of missing values was performed via nuclear-norm regularisation implemented in the *softImpute* [49] R package. Cross validation (5-fold) ROC-AUCs and accuracy were computed to assess the performance of the model. A set of 33 genes externally assessed as EL [50] was used as additional validation (File S2 [33]).

### Gene similarity approach

Similarity with known genes associated to biallelic forms of inherited metabolic disorders (biallelic inborn error of metabolism green genes from PanelApp, BIEM) was assessed according to 5 attributes (5ps): (p1) being a paralogue of a known BIEM gene according to Ensembl genes 104 and a threshold of % amino acid identity of 20% [41]; (p2) sharing a Reactome pathway (lowest level) with a BIEM gene [43]; (p3) belonging to the same Corum protein complex of a BIEM gene [45]; (p4) being a direct interactor within the protein-protein interaction network (high confidence cut-off 0.7) of a BIEM gene according to STRING [42]; and (p5) sharing a PFAM protein family with a BIEM gene [44]. The number of features shared was computed for every early lethal gene—assessed and predicted (File S3 [33]).

### Investigation of cases from the 100KGP

To investigate the occurrence and enrichment of homozygous LoF variants in cases from the 1000KGP among our set of EL genes in the mouse, we searched for variants in those genes in 35,422 families, 631 of which were recruited under the categories of interest (‘undiagnosed metabolic disorders’ and ‘mitochondrial disorders’). One important caveat is that these are not healthy population controls, and we cannot rule out that patients recruited under other categories show similar metabolic phenotypes, which means that these ratios can be an underestimation. The number of observed homozygous LoF and missense variants prioritised by Exomiser based on variant scores [20] were compared between cases and *pseudo* controls to compute observed versus expected ratios (File S4) [33].

### Statistical analysis and software

R software [51] including the following packages were used for data integration and analysis: *tidyverse* [52], *matrixStats* [53], *epitools* [54], *DescTools* [55], *oddsratio* [56]; data visualisation: *waffle* [57], *ggridges* [58], *alluvial* [59], *cowplo*t [60], *upSetR* [61]; ontologies: *ontologyIndex* [62]; modelling and prediction: *softImpute* [49], *gamsel* [48], *pROC* [63]. To test for significant differences in the proportions of cellular essential genes, genes with no paralogues, paralogues properties, Mendelian disease genes, modes of inheritance and disease categories across the 3 WoL and to perform pairwise comparisons, Pearson’s chi-squared (two-sided) was used as implemented in *prop.test* and *pairwise.prop.test* functions. In the case of continuous variables, to test for significant differences between the three WoL, we used the non-parametric Kruskal-Wallis test to compare the three groups and post hoc Dunn’s test for pairwise comparisons (two-sided) using their R implementations*: kruskal.test* and *dunnTest* functions. The null hypotheses being that the distribution of the CERES depletion scores for the different tissues, the levels of gene expression across development and several intolerance to variation metrics is the same across WoL. For each cell lineage, all the gene individual scores were used to assess statistical significance. 95% CI for the median score for each window and cell line were computed using the exact method implemented in the *MedianCI* function in the *DescTools* R package. The thresholds for statistical significance after multiple testing corrections are specified in Additional file 1: Tables S1-S4. Odds ratios (OR) were calculated by unconditional maximum likelihood estimation (Wald) and confidence intervals (CI) using the normal approximation, with the corresponding adjusted *P* values (Benjamini-Hochberg, BH) for the test of independence using the *oddsratio* function (Additional file 1: Table S5). To evaluate the performance of our approach to identify candidate genes, *F*-scores were computed for our strategy based on EL genes and alternative ones based on pRec, DOMINO, SCoNeS and LOEUF scores. Precision and recall were estimated based on the number of predicted recessive genes using the suggested thresholds for the different scores and the number of BIEM genes in each of these sets of candidate genes. A multiple logistic regression model was fitted using EL and these other metrics as predictors of BIEM genes and the ORs associated with each predictor for specific increment steps were estimated as implemented in the *or_glm* function (Additional file 1: Tables S6-S7).

## Results

### Gaining functional knowledge from WoL

**Fig. 1:**
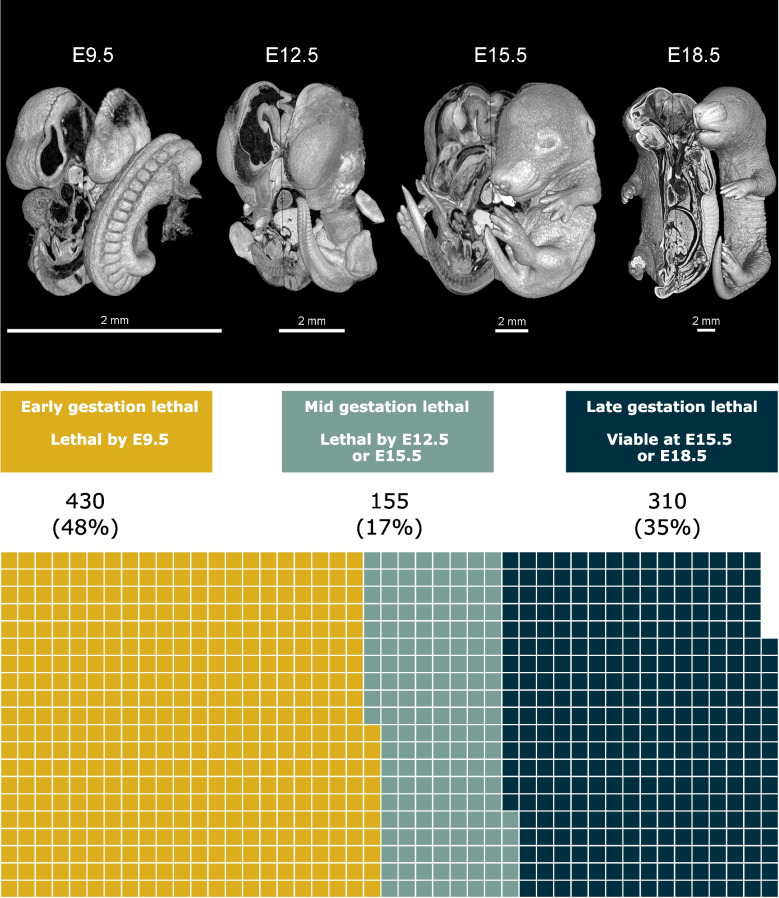
Description of WoL and distribution of lethal genes across these windows. Three-dimensional microCT images of wild-type mouse embryos corresponding to E9.5, E12.5, E15.5 and E18.5. The waffle chart shows the total number of lethal genes characterised through the secondary viability screening and their distribution by WoL. EL genes, where the embryo dies before E9.5 constitute nearly 50% of all the lethal genes in this dataset. This stage broadly correlates with the pre-organogenesis phase of embryonic development. Non-early lethal genes are divided into ML (17%) and LL (35%). The complete set of genes associated with each WoL is available in File S1 [33]. WoL, windows of lethality; E, embryonic day; EL, early gestation lethal; ML, mid gestation lethal; LL, late gestation lethal

The IMPC measures viability between 14 days of age and weaning and, for lethal strains, employs a high-throughput embryonic phenotyping pipeline to examine embryo viability and phenotypes at embryonic day (E) E9.5, E12.5, E15.5 and/or E18.5. The developmental period during which lethality occurs in the mouse can be summarised by establishing a set of WoL. A WoL for a gene was defined by the interval between the latest developmental stage at which live homozygous null embryos (mice) are identified and the earliest stage at which no live homozygous embryos are found [24]. Complete lethality by E9.5 was classified as early-gestation lethal (EL), by E12.5 or E15.5 as mid-gestation lethal (ML), and viability at E15.5 or E18.5 as late-gestation lethal (LL). These WoL approximately correlate with the pre-organogenesis, organogenesis and post-organogenesis phases of mouse embryonic development, while also providing sufficient sample sizes to perform downstream statistical analyses. Among 895 embryonic lethal genes with one-to-one human orthologues assessed in the IMPC to date, nearly half (430, 48%) are EL, 155 (17%) ML, and 310 (35%) are LL. A full description of the WoL is available (File S1 [33]) and the distribution of lines per window can be found in Fig. 1.

#### Human cellular essential genes correlate with mouse EL genes

**Fig. 2:**
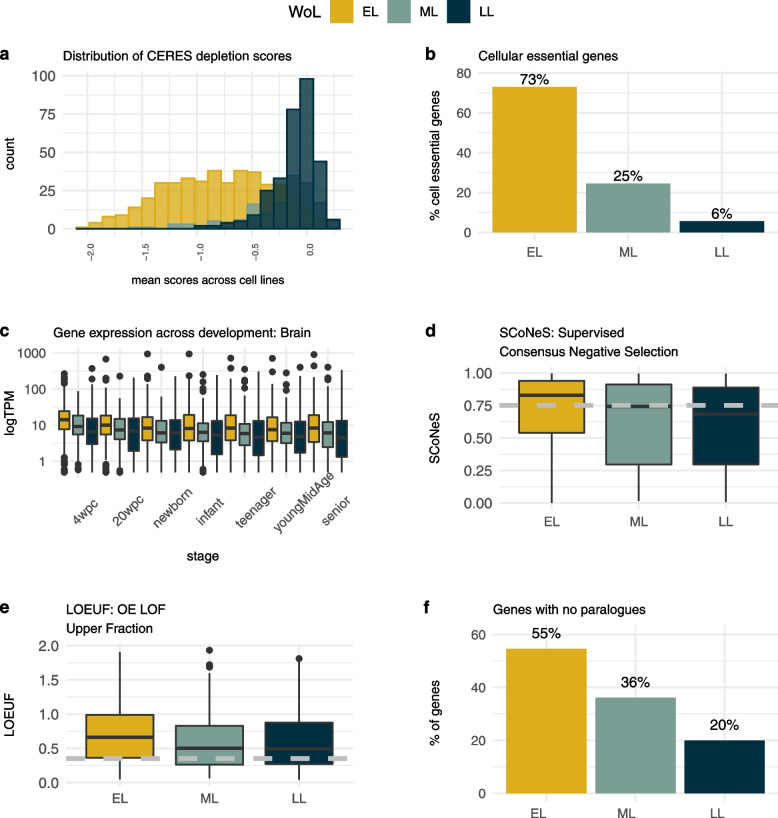
WoL and gene features. **a** Distribution of mean CERES depletion scores. Histograms represent the probability distribution of mean CERES scores across cell lines for each WoL. **b** WoL and cellular essential genes. Percentage of EL, ML and LL genes considered cellular essential when a mean CERES depletion score across cell lines of −0.45 is considered as threshold. **c** Gene expression in brain. Boxplots show the distribution of human gene expression values for genes within each WoL across selected developmental stages for human brain. **d** SCoNeS scores. Boxplots show the distribution of SCoNeS scores, the predicted probability of a given gene being AR. The dashed grey line represents a threshold (SCoNeS > 0.75) used to identify genes underlying AR disorders. **e** LOEUF scores. Boxplots show the distribution of LOEUF scores across WoL. Low LOEUF scores indicate strong selection against predicted loss-of-function (pLoF) variation in a gene. The dashed grey line represents a threshold (LOEUF <0.35) used to identify genes that are constrained against pLoF variation. **f** WoL and paralogues. Barplots represent the percentage of genes with no paralogues (singletons) across WoL, with the proportion of genes with no duplicates decreasing across development stages. Tests for differences between WoL available in Additional file 1: Table S1-S3. For plots **a–f**, the data shown correspond to gene annotations for the human orthologues. WoL, windows of lethality; EL, early gestation lethal; ML, mid gestation lethal; LL, late gestation lethal; LOEUF, LoF observed/expected upper bound fraction; SCoNeS, supervised consensus negative selection; AR, autosomal recessive

We previously reported that EL genes show a considerable overlap with human cellular essential genes [25]. The CERES dependency scores obtained from CRISPR knockout screens through the Achilles pipeline [35] compute the depletion effect on cell proliferation. A lower and more negative value is the result of greater depletion of cancer cells upon genetic perturbation and indicates higher essentiality [64]. Plotting median proliferation scores and the corresponding 95% CI of genes for different human cell lines across tissues, we observed a clear distinction between the three WoL. The set of EL genes stands alone as a distinctive category from the ML and LL genes that have closer median values (Additional file 2: Fig. S1). The differences in score distribution are consistent and statistically significant across cell lineages (*P* value < 2.2e−50), with a few exceptions when comparing ML and LL sets (Additional file 1: Table S1). Considering the average CERES score across 902 cell lines, we observed that only EL genes are found in the bins with lowest scores, and that the percentage of ML and LL genes within bins increased with higher values of this score (Fig. 2a, Additional file 2: Fig. S2a). When cellular essentiality is considered as a binary property after categorising the mean scores using a cut-off of −0.45 (≤ −0.45: ‘cellular essential’, >−0.45: ‘cellular non-essential’; see the ‘Methods’ section), 73% of EL genes are essential in human cell lines, compared to 25% of ML genes and only 6% of LL genes (*P* value < 2.2e−50) (Fig. 2b, Additional file 1: Table S1). Alternative thresholds are considered in Additional file 2: Fig. S2b-2c and show a similar enrichment. Cell line essentiality was previously explored for mouse viable genes and showed that > 99% are non-essential in human cell lines [25]. We additionally examined individual cell lines to discard any potential cell line specific effect, and the percentage of EL genes found to be essential in each cell line based on this threshold ranges from 58 to 79% with a mean value of 72% (ML mean 24%, range 15–34%; LL mean 9%, range 5–25%).

#### EL genes consistently show higher levels of human gene expression across developmental stages

Examination of human gene expression data [36] showed a consistent pattern of expression in brain across developmental stages with the human orthologues of mouse EL genes being expressed at higher levels, on average, compared to the orthologues of mouse ML and LL genes, and with the differences in gene expression between EL and LL genes being statistically significant across most developmental stages (Fig. 2c, Additional file 2: Fig. S3a, Additional file 1:Table S2). A similar pattern was observed for other organs with data available, including cerebellum, heart, kidney, liver, ovary and testis (data not shown). High levels of expression may help identify key developmental processes. To that end, gene expression patterns during early human development have been used to predict essential genes lacking a known human disease association [65]. To assess whether the organ development trajectories for these genes differ substantially between mouse and human, we investigated the similarity of spatiotemporal gene expression profiles for the two species. We found that 78 and 82% of the entire set of genes studied showed the same trajectory for cerebellum and brain respectively, with no significant differences observed between WoL and in concordance with what was observed for the entire set of genes with data available [37] (Additional file 2: Fig. S3b). Similarities in gene expression do not always imply conserved phenotypes between mouse and human, but can serve as a proxy for how translatable the findings for these genes are to human disease.

#### Intolerance to LoF variation differs across WoL

EL genes are more likely to underlie an AR condition, based on higher Supervised Consensus Negative Selection scores (SCoNeS) [39], a metric that estimates the predicted probability of a gene being AR, particularly when compared to LL genes (unadjusted *P* value = 5.45e−07; Fig. 2d). When the LoF observed/expected upper bound fraction (LOEUF) [13], a quantitative measure of the observed depletion of LoF variation compared to a null mutational model, was investigated, we observed an inverted pattern, with EL genes showing higher mean values of this score (weaker selection against predicted LoF variants) compared to ML and LL genes (unadjusted *P* values 6.70e−03 and 2.69e−04 respectively; Fig. 2e). Albeit only nominally statistically significant, this observation agrees with our previous findings that developmental lethal genes, those genes that are not essential for cell survival but required for organism development, and that broadly correlate with ML or LL genes, are more intolerant to heterozygous LoF variation compared to cellular lethal genes, those found to be essential in human cell lines and lethal in the mouse, and more likely to be EL [25]. Additional constraint metrics were explored, including pLI and pRec [13], RVIS [40] and DOMINO [38] (Additional file 2: Fig. S3c-3f). DOMINO scores represent a gene level metric based on a machine learning approach that extracts discriminant information from a broad set of features and computes the probability for a gene to carry dominant mutations. Based on this measurement, EL genes were also more likely to be linked to AR disease compared to LL genes (unadjusted *P* value = 3.39e−05; Additional file 2: Fig. S3g). The results for the statistical tests of significance are shown in Additional file 1: Table S3.

#### Gene duplicates and time of duplication event are distinctive features of EL genes

EL genes have the highest proportion of genes with no paralogues (singletons). This proportion decreases gradually from ML to LL genes (unadjusted *P* value = 1.41e−20; Fig. 2f). Not only are EL genes more likely to be singletons, but also, for those genes that do have paralogues, the number of paralogues is lower and the paralogues are more likely to be older, with longer times since the duplication event when compared to ML or LL genes, which suggests more time to evolve new functions (Additional file 2: Fig. S4a, S4b). Thus, not only do gene duplications, or the lack thereof, seem to play a role in essentiality but so do the number of paralogues and the time of the duplication event. Similar observations were made by others using different species and/or definitions of essentiality [66, 67]. Paralogues of EL genes are also more likely to be EL, and similarly paralogues of ML/LL genes are more likely to be ML/LL (Additional file 2: Fig. S4c). This implies that paralogues are predominantly essential at the same developmental stage, potentially reflecting similar key functions at the cellular level and early stages of organism development. The differences in all these metrics are statistically significant when comparing EL vs LL genes (Additional file 1: Table S3). Additionally, by dividing genes into singletons and duplicates, we explored the proportion of genes that are cellular essential among these two sets of genes for the three WoL (Additional file 2: Fig. S4d). Previous studies investigating the relationship between essentiality, developmental expression and gene duplication have suggested that timing of developmental expression influences the ability of a gene in a paralogue pair to compensate for the loss of function of the other gene [68].

### WoL and Mendelian disease

It is well established that there is an association between lethal genes in the mouse and human disease genes [24, 47]. Our previous study showed that this enrichment was mainly driven by developmental lethal genes [25] so we hypothesised that the distribution of disease genes across WoL may not be uniform and that information about WoL could reveal additional correlations. When translating our WoL to relevant developmental stages in humans, the EL mouse category broadly correlates with the human pre-organogenesis stage occurring during the first 2 weeks of development. The ML class relates to human organogenesis occurring during the embryonic period from weeks 3 through 8, and ending in the first trimester, around week 9 of gestation. Lastly, the LL category aligns with the human foetal stage, from week 9 until birth [69].

We used PanelApp as the source of Mendelian genes to perform subsequent analyses [9]. Genes are rated according to level of evidence to support the phenotype association: ‘green’ means high level of evidence from several unrelated families and/or strong additional functional data, ‘amber’ moderate evidence and ‘red’ not enough evidence. The advantages of using this source of diagnostic genes include the high-level disease categorisation and allelic requirement annotations that allows for tailored analysis, the categorisation of genes according to the level of evidence for the gene-disease association and the potential to map directly to patient data recruited in the 100KGP.

#### Disease category and mode of inheritance are not uniformly distributed across WoL

**Fig. 3:**
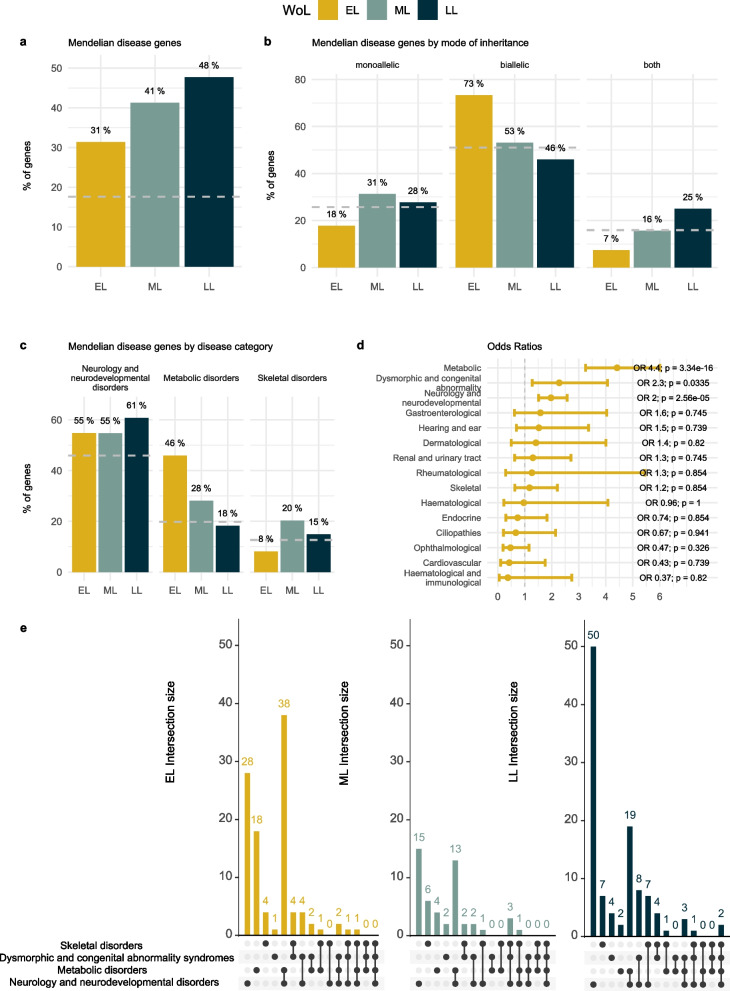
WoL and human disease. **a** Mendelian disease genes. Barplots represent the percentage of rare disease associated genes in each WoL according to PanelApp, only ‘green’ genes with a high level of evidence for the gene-disease association were included. **b** Mode of inheritance. Barplots represent the percentage of Mendelian genes by associated allelic requirement across WoL, only monoallelic or biallelic genes were included. **c** Disease category. Mendelian genes by disease type according to PanelApp level 2 disease categories, with the bars indicating the percentage of PanelApp genes mapping each disease class for the 3 WoL. For plots **a**–**c**, the dashed grey line represents the baseline percentage for the entire set of protein coding genes (19,197 genes according to HGNC, **a**) or PanelApp ‘green’ genes (3384 genes, **b**, **c**). **d** Disease categories OR and BH adjusted *P* values for EL genes compared to ANEL genes: this included mid and late gestation lethal genes as well as subviable and viable categories. **e** Disease category overlap. Overlap between genes associated with the most frequent disease categories across WoL for EL, ML and LL genes respectively. Tests for differences between WoL are available in Additional file 1: Table S4. WoL, windows of lethality; EL, early gestation lethal; ML, mid gestation lethal; LL, late gestation lethal; HGNC, HUGO Gene Nomenclature Committee; ANEL, all non-early gestation lethal genes; OR, odds ratio; BH, Benjamini-Hochberg

Although the three WoL are all enriched for Mendelian disease genes, their properties differ. The proportion of genes associated with rare disorders is lowest among the EL, followed by the ML and LL genes (Fig. 3a). When allelic requirement is considered, this trend is reversed for AR disorder-associated genes, where the EL fraction showed a significantly higher number of biallelic genes compared to LL genes (unadjusted *P* value = 5.16e−06; Fig. 3b; results for the statistical tests of significance in Additional file 1: Table S4).

Further dissection of disease genes according to PanelApp high level disease categories showed that (1) the proportion of neurodevelopmental disorder associated genes is higher than expected among the three WoL compared to baseline, with the highest percentage among LL genes; (2) the proportion of genes associated to metabolic disorders follows the inverse pattern, with EL genes showing the highest percentage of inherited metabolic disease genes (46%), followed by ML (28%) and showing the lowest percentage among the LL (18%) (unadjusted *P* value = 2.7e−06); most notably, this is the only disease category with a higher percentage of disease genes among the EL compared to ML and LL genes; (3) a higher percentage of skeletal disorder genes is found in ML set, although this association is only nominally significant; and (4) for the remaining disease categories, the frequency of disease genes among the EL genes shows values comparable to baseline or even lower, indicative of depletion of these disease categories among the EL genes (Fig. 3c, Additional file 2: Fig. S5a, Additional file 1: Table S4). In order to assess the strength of the association between EL genes and the different disease categories, OR were computed using the entire set of non-EL genes, i.e. all those genes with IMPC data on viability, including ML, LL, subviable and viable categories (see the ‘Methods’ section). Three disease categories showed a positive association (with a lower bound of the 95% CI for the OR > 1): metabolic disorders (OR = 4.4; adjusted *P* value = 3.34e−16), dysmorphic and congenital abnormality syndromes (OR = 2.3; adjusted *P* value = 0.034) and neurology and neurodevelopmental disorders (OR = 2; adjusted *P* value = 2.56e-05) (Fig. 3d).

Given that most inborn errors of metabolism (IEM) show neurological manifestations, and neurodevelopmental disorders are still the most predominant disease category across the three WoL, we further explored the gene overlap between neurodevelopmental and metabolic disease categories to assess any potential confounding effect. The combination of genes associated with both metabolic and neurodevelopmental disorders was found to be predominant among the EL class, opposite to what we observed among the ML and LL classes, where neurodevelopmental only genes are the prevalent disease class, thus providing additional evidence for the IEM association with EL genes (Fig. 3e).

The analysis of HPO phenotypes associated with known inborn error of metabolism genes showed that the five most frequent physiological systems affected are nervous system, followed by musculoskeletal, metabolism/homeostasis, growth abnormality, and digestive. An enrichment analysis showed no significant differences in the frequency of any of these phenotypes among EL genes when compared to ML and LL genes (Additional file 2: Fig. S5b, Additional file 1: Table S5).

#### Evidence of prenatal and perinatal lethality in humans

Among the wide range of Mendelian phenotypes observed in humans, prenatal lethality poses a unique challenge in terms of providing a molecular diagnosis. Development failure may occur at any point between fertilisation and birth. Estimates suggest that 20–30% of implanted embryos fail to develop beyond week 6 [70]; similarly early embryo losses occurring between implantation and clinical recognition could be around 10–25% [71]. A proportion of first trimester miscarriages where no chromosomal abnormalities are detected could have a Mendelian or polygenic origin [72, 73].

We previously hypothesised that many human genes contributing to prenatal lethality are likely unidentified and not captured in current disease databases due to early embryo losses and miscarriages either being unnoticed, or when they are detected, the difficulty in determining the molecular basis of this extreme phenotype. Here, we used a set of 624 genes associated with early lethality in humans curated from OMIM [6, 47]. We found that 19% of EL disease-associated genes are linked to pre- and perinatal lethality. For LL genes, this percentage is 31% (Additional file 1: Fig. S5c). Based on our hypothesis that most genes associated to early-gestation lethality in humans remain unrecognised, the set of EL genes in the mouse constitutes a source of candidates of interest in the field of foetal precision medicine.

#### Predicting new EL genes in the mouse

Since the number of IMPC mouse lines that have undergone the primary viability assessment is higher than those with a secondary evaluation to identify the embryonic stage at which lethality occurs, we tried to predict additional EL genes among lethal genes without secondary viability data to have a larger pool of candidate genes. For this, we used a penalised likelihood approach to fit a generalised additive model using proliferation (essentiality) scores from multiple human cell lines as predictors [35] and subsequently used that model to make the predictions. This added a further set of 362 predicted EL genes (out of 725 lethal genes with no secondary viability assessment) to the previous 430 EL genes assessed through embryo viability screening. Details on the model, predictive accuracy, and predictor variables are described in the ‘Methods’ section and Additional file 2: Fig. S6. Of 33 genes in our prediction set that were externally assessed as EL [50], 29 were correctly predicted by the classifier (87.9%) [33]. CRISPR knockout screens to identify those genes affecting cell survival across hundreds of genomically characterised cancer cell lines [74] can consequently assist with the identification of early-gestation lethal lines in the mouse.

#### Similarity with known BIEM genes

**Fig. 4:**
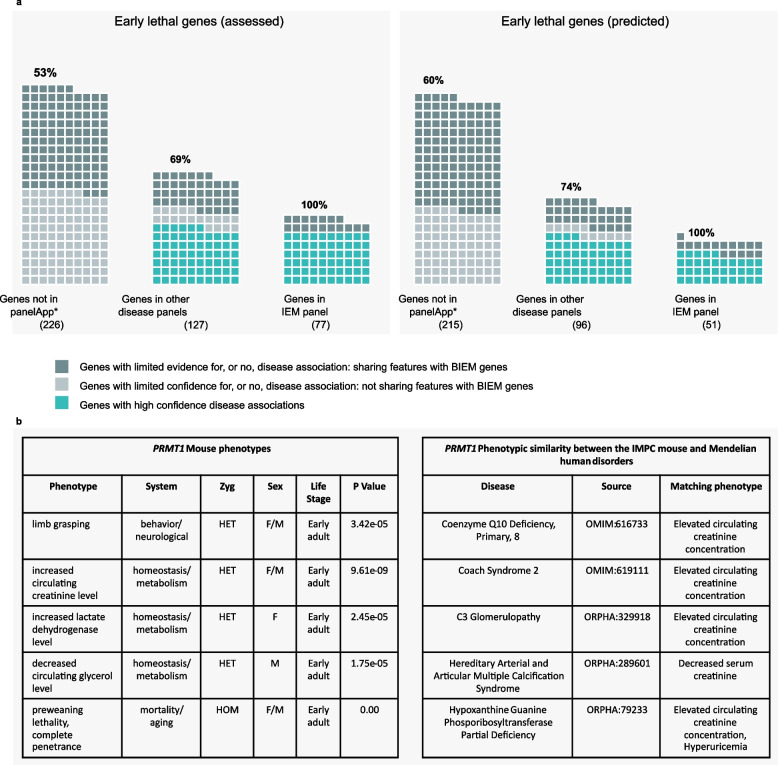
Gene similarity approach. **a** Genes sharing features with BIEM genes for each category of EL genes based on evidence for the gene-disease association. Each set of EL genes in the mouse (assessed and predicted) is broken down into 3 sub-categories based on PanelApp evidence: genes associated with inborn errors of the metabolism, Mendelian disease genes in other disease categories and non-disease genes. For genes in PanelApp panels, the genes are also subdivided into those with strong evidence for the gene-disease association (green) and those with more limited evidence to date (red or amber). The percentage of genes sharing one of the 5 features (paralogue, protein family, ppi, pathway, protein complex) with known BIEM genes is shown for potential novel genes absent from PanelApp as well as those with more limited evidence (red or amber). For each sub-category, those genes sharing ≥4 features with known BIEM genes are shown. Nine assessed and 5 predicted EL genes that are included in this figure as amber/red genes in the IEM panel are also green genes in other disease panels (see Files S3-S4 [33]). **b*** PRMT1* IMPC mouse evidence. Mouse phenotypes and phenotypic similarity with human disorders. Heterozygous knockout phenotypes include several metabolic and neurological abnormalities. When computing the similarity between the mouse and human disease phenotypes associated with known disorders, we find phenotypic overlap with several early onset conditions, including defects of the metabolism coenzyme Q10 deficiency, primary, 8 and hypoxanthine guanine phosphoribosyltransferase partial deficiency. EL, early gestation lethal; BIEM, biallelic inborn errors of the metabolism; ppi, protein-protein interaction; IMPC, International Mouse Phenotyping Consortium

A gene similarity strategy was applied to 792 (assessed and predicted) human orthologues to mouse EL genes based on features shared with 552 diagnostic-grade BIEM genes from PanelApp. This approach was based on the unknown gene sharing at least one of 5 attributes: (p1) being a paralogue of a known BIEM gene; (p2) sharing a pathway with a BIEM gene; (p3) belonging to the same protein complex as a known BIEM gene; (p4) interacting with a known BIEM gene; and/or (p5) sharing a PFAM protein family with a known BIEM gene. This gene ranking approach served a dual purpose: (1) to identify completely novel disease genes and (2) to bring additional proof for those genes in PanelApp that are not considered diagnostic-grade genes, i.e. ‘amber’ and ‘red’ genes. Among novel EL genes not associated with any disease in PanelApp, 53–60% share at least one of the above five attributes with a BIEM gene. This percentage increases to 69–74% when the non-diagnostic-grade genes in PanelApp excluding the IEM panel are examined and to 100% for the non-diagnostic-grade genes on the IEM panel (Fig. 4a).

Ten of the EL non-disease-associated genes are of particular interest as they share 4 of the 5 attributes with BIEM genes: *CHKA*, *FDX1*, *GGPS1*, *GLRX3*, *HMGCS1*, *MGAT1* and *SLC39A10* are paralogous and direct interactors as well as belonging to the same protein family(ies) and pathway(s) while *MRPS25*, *PRMT1* and *RPA1* are interactors, share a protein family(ies) and pathway(s) and are also part of the same protein complex(es). The complete gene list and annotations are provided in [33]. Four of these genes, *Ggps1*, *Mrps25*, *Prmt1* and *Rpa1*, show abnormal metabolic phenotypes in the heterozygous viable mouse [31]. *MRPS25* is a member of the human mitochondrial ribosomal protein gene family, with evidence from mouse embryos indicating compromised mitochondrial function [75]. Several other mitochondrial ribosomal small (MRPS) and large (MRPL) subunit genes are associated with different metabolic disorders, and many of the remaining MRPS genes are also potentially associated with disease [76]. Evidence of pathogenicity of homozygous missense variants in this gene has been reported [77]. In the case of *PRMT1*, encoding a member of the protein arginine N-methyltransferase (PRMT) family, additional neurological phenotypes found in the IMPC knockout of the orthologous *Prtm1* imply a high phenotypic similarity with neonatal disorders including several defects of the metabolism as computed by PhenoDigm [32] (Fig. 4b). Emerging evidence supports the role of this family of enzymes in skeletal muscle and metabolic disease [78].

To evaluate this approach, and whether EL genes not associated with Mendelian disorders are more likely to share attributes with BIEM genes compared to non-EL and non-disease associated genes, we computed the ORs to obtain a measure of this association. Importantly, we found a significant association between sharing any of these 5 attributes with a BIEM gene and being EL (1.64 fold-increase, adjusted *P* value = 2.7e−06). When these attributes were considered separately, the strongest association was observed for being part of the same protein complex as a BIEM gene (13.9 fold-increase, adjusted *P* value = 6.5e−20). Significant results were also obtained for sharing a pathway and interacting with a BIEM gene. EL genes were less likely to be a paralogue of a BIEM gene (OR = 0.49, adjusted *P* value = 0.018), which can be explained by the enrichment for singletons among this set of genes (Additional file 2: Fig. S7).

Disaggregating the set of EL genes by disease association showed that the closer to the IEM disease class, the higher the percentage of genes in that category sharing attributes with BIEM genes. Consistently, EL genes are more likely to share attributes with BIEM genes compared to non-EL genes.

#### Undiagnosed cases of inherited metabolic disorders from the 100KGP

An alternative approach, based on patient data, was also used to identify potential metabolic disease genes among the set of EL genes in the mouse. Cases recruited under the ‘undiagnosed metabolic disorder’ and ‘mitochondrial disorders’ categories in the 100KGP were investigated for rare, segregating and biallelic LoF or predicted pathogenic missense variants in EL genes, using the Exomiser variant prioritisation tool [20]. Observed versus expected (OE) ratios per gene were computed by comparing the number of biallelic variants observed in these patients to those observed on a set of *pseudo controls*, i.e., patients recruited under other disease categories. Predicted homozygous or compound heterozygous pathogenic variants were found in 21 EL genes (13 assessed, 8 predicted) with OE ratios > 1 and observed in ≤ 2 controls. None of the 21 genes showed enrichment of synonymous variants by these same criteria. Out of the 21 genes, 3 involved biallelic LoF, 6 had biallelic LoF/missense and 12 had biallelic missense variants. Five of these genes are already classified as diagnostic grade genes in the IEM panel (*COQ4*, *ELAC2*, *MRPL44*, *MSTO1* and *SKIV2L*) and three others are diagnostic grade genes in different neurology and neurodevelopmental disorder gene panels (*EIF2B4*, *ELP1*, *EXOSC8*). *ALG2*, *NDUFA8* and *RNASEH2A* are classified as amber or red in the IEM panel. For the cases associated with these 11 known disease genes, only those associated with *MRPL44* and *ALG2* biallelic variants have been diagnosed with these variants so far, with the others currently classified as variants of uncertain significance. For the remaining 10 genes (*AFDN*, *CDK12*, *COQ3*, *GINS4*, *GPATCH1*, *INTS11*, *KIF2C*, *NUFIP1*, *PTPMT1*, *RCC1*), there is no current evidence for a disease association in PanelApp or OMIM. The complete set of genes is provided in File S4 [33].

For two of the amber or red genes in the IEM panel, *ALG2* and *NDUFA8*, IMPC heterozygous knockout mice have neurological and metabolic phenotypes [31], providing additional evidence to validate this gene-disease association. In addition, *ALG2* shares 4 features with known BIEM genes: protein family (2 genes), pathway (10 genes), paralogue (1 gene) and protein-protein interaction (9 genes). Similarly, *NDUFA8* shares 3 features: protein complex (17 genes), pathways (44 genes) and protein-protein interaction (28 genes).

**Fig. 5:**
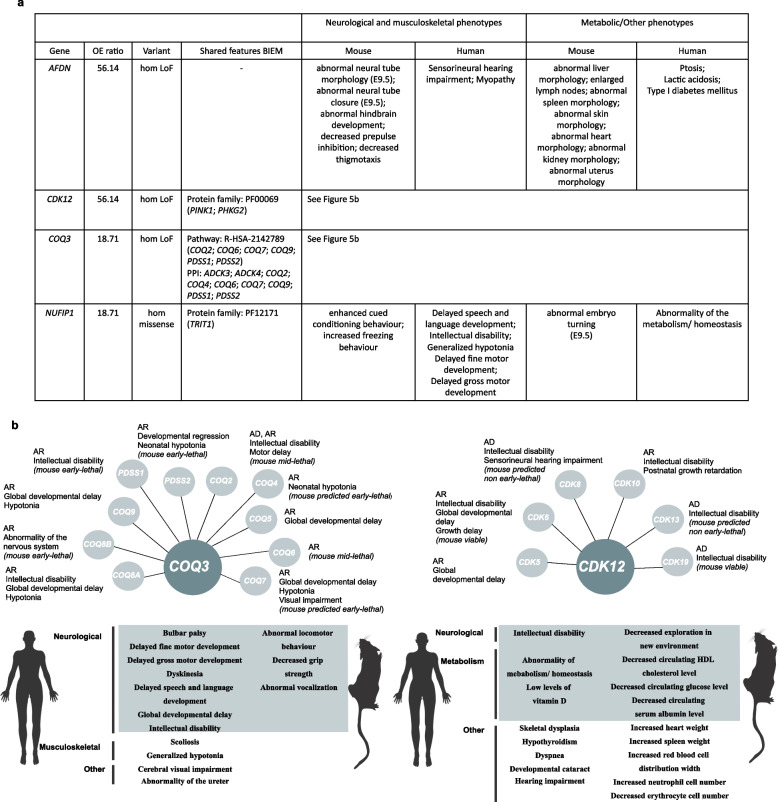
Candidate genes with biallelic inheritance involving LoF or (missense) predicted pathogenic variants in undiagnosed patients. **a** Mouse evidence. Genes with homozygous LoF or missense variants found in patients recruited under the ‘undiagnosed metabolic disorder’ and ‘mitochondrial disorders’ disease categories with an OE ratio > 1, observed in ≤ 2 controls and with the IMPC heterozygous knockout mouse displaying abnormal phenotypes in the relevant physiological systems, partially mimicking the phenotypes observed in patients. **b*** COQ3* and *CDK12* belong to families and pathways with several genes associated with Mendelian disorders. The corresponding mode of inheritance and related/overlapping phenotypes for these known disease associated genes and evidence on viability from the IMPC are shown. Information on prioritised genes available in File S4 [33]. LoF, loss-of-function; OE, observed vs expected; IMPC, International Mouse Phenotyping Consortium; AD, autosomal dominant; AR, autosomal recessive

Four non-disease-associated genes have IMPC data for null alleles with heterozygous mouse mimicking some of the clinical features observed in patients. *AFDN* and *NUFIP1* show neurological phenotypes in the orthologous mouse embryo or early adult [31, 32]. *COQ3* and *CDK12* also show neurological and other physiological system phenotypes [31, 32] shared between the undiagnosed patients and the knockout mouse. Detailed information on the phenotypes observed in the patients is shown in Fig. 5a, b. They are of particular interest as several other genes from the same family have already been associated with similar disorders, and the IMPC lines are the first reported mouse models with abnormal phenotypes observed in the early adult heterozygous knockout [79].

*COQ3* (coenzyme Q3, methyltransferase) is one of the genes required for the biosynthesis of Coenzyme Q10, which has many vital functions. Several genes involved in this pathway are associated with Primary CoQ10 Deficiency, including *PDSS1*, *PDSS2*, *COQ2*, *COQ4*, *COQ5*, *COQ6*, *COQ7*, *COQ8A*, *COQ8B* and *COQ9* [80]. The heterozygous *Coq3* IMPC mouse shows several neurological/behavioural phenotypes including abnormal locomotor behaviour, abnormal vocalisation and decreased grip strength. No homozygous LoF variants have been observed for this gene according to gnomAD (pLI = 0; pRec = 0.283; DOMINO = very likely recessive). The homozygous frameshift variant observed in the 100KGP cohort is present in gnomADv2.1.1 (p.Lys366SerfsTer2), with an allele frequency of 6.04e−04 but with no homozygous individuals for that allele. The OE ratio in our 100KGP study cohort is 18.7, with the other two different variants found in the set of *pseudo controls* recruited under the ‘unexplained sudden death in the young’ and ‘ultra-rare undescribed monogenic disorders’.

C*DK12* (cyclin dependent kinase 12) is one of the cyclin-dependent kinases with a key role in molecular processes relevant during development. Several other protein kinases are involved in developmental disorders: *CDK5*, *CDK6*, *CDK8*, *CDK10*, *CDK13* and *CDK19* [81]. The phenotypic abnormalities observed in heterozygous *Cdk12* IMPC mice include cardiac, haematopoietic, metabolic (decreased circulating HDL cholesterol level) and neurological features (decreased exploration in new environment) (Fig. 5b). The homozygous splice acceptor variant (c.1047-2A>G) is present in gnomADv2.1.1, with an allele frequency of 4.06e−4 and one homozygote observed in the South Asian population. This gene is in fact predicted to be highly intolerant to heterozygous LoF variation (pLI = 1; pRec = 0; DOMINO = very likely dominant). The OE ratio computed with biallelic variants in our GEL study cohort for this gene is 56.14 with no variants meeting the criteria described found in controls.

A note of caution is needed when interpreting the impact of these two homozygous LoF variants in *COQ3* and *CDK12* identified in the 100KGP cohort due to their position on the transcript (near the end of the transcript and into a NAGNAG sequence, which may indicate a frame-restoring splice site, respectively), as indicated by gnomAD. Where available, data on gene expression across development for the aforementioned genes (*AFDN*, *NUFIP1*, *COQ3* and *CDK12)* confirmed similar developmental gene expression profiles across time points from early organogenesis to adulthood in brain and cerebellum between mouse and human, which supports the translatability of the findings in the knockout mouse for these genes [37].

## Discussion

Many predicted LoF variants identified in Mendelian disease sequencing studies are found in genes not previously associated with disease, making assessment of pathogenicity particularly challenging. High-throughput mouse standardised phenotyping screens including viability assessment contribute to acquiring new knowledge about orthologues of such genes with limited functional data [82, 83]. By also exploring correlations between abnormal phenotype(s) in the knockout mouse and disease features in the human orthologues, we were able to identify novel candidates for Mendelian conditions.

Previously, we developed a successful framework to prioritise gene candidates for neurodevelopmental disorders using mouse phenotyping data, with two of the top nine candidate genes, *VPS4A* and *SPTBN1*, having been recently validated. In both cases, a causal link has been found between heterozygous, predominantly de novo mutations and distinctive developmental syndromes [25–27]. Here we present another example of how the IMPC data resource can be combined with other sources of evidence to develop a tailored approach for disease-gene discovery and variant prioritisation to assist the diagnosis of inherited metabolic disorders.

The requirement of a gene for the survival of an organism, i.e. gene essentiality, can be disaggregated into more granular categories/WoL according to the embryonic period during which lethality occurs. In the present study, we show that these categories correlate with different gene features, including gene expression across development and intolerance to LoF variation. Higher levels of gene expression among cellular essential genes compared to non-essential genes have been previously reported across developmental stages [84]. Human embryonic gene expression data, integrated with other gene features has been used to identify essential genes, suggesting that gene-specific expression changes during early development could be particularly relevant [65]. Importantly, housekeeping genes, defined as those genes being stably expressed irrespective of tissue and developmental stage, are not necessarily essential, and the genes that are both essential and invariably expressed may differ across organisms [85]. Additionally, the distribution of singleton and duplicated genes across these WoL supports hypotheses about the ability of paralogues for functional compensation at the cellular level [86]. EL genes are more likely to be singletons, and when paralogues exist, they tend to have originated earlier, suggesting more time to evolve new and/or distinct functions [66, 67]. Paralogue functional compensation is not a universal ability, and physical and functional dependencies of the paralogues could reduce their buffering capacity [87]. Studies of synthetic lethality between paralogue pairs suggest which gene features may be associated with the ability to compensate for each other’s function [88].

By looking at different features of human orthologous disease genes across the WoL, two observations stand out. First, the set of lethal genes in the mouse is enriched for Mendelian disease genes [24], but the proportion of genes associated with disease is not consistent across WoL with this enrichment mainly driven by LL genes. The lower proportion of disease genes among the EL compared to LL genes was previously reported when comparing cellular lethal with developmental lethal genes [25], as well as other categorisations of essential genes [47, 89]. Second, we identified a strong association between EL genes and inherited metabolic disorders. This includes genes that are needed to maintain the metabolic machinery required to provide energy and basic components for cell survival. Most of the EL lines die prior to implantation or gastrulation, and differentiation into disease-associated tissues occurs at a later stage. This could explain why non-metabolic disease categories are underrepresented among the set of EL genes.

Building on this finding, we focused on the EL genes and gathered additional information on similarity with known disease genes associated with BIEM disorders. It is already known that members of paralogous gene families where one gene is associated with human disease are more likely to be associated with Mendelian disorders themselves [90]. Similarly, disease-associated variants are enriched at sites conserved among paralogues [91, 92]. We used these and other observations to identify the EL genes showing most similarity to existing BIEM genes and, hence, most likely to be novel BIEM disease genes.

Inherited metabolic disorders comprise a large group of ~1450 disorders in which the primary alteration of a biochemical pathway leads to a set of biochemical, clinical and/or pathophysiological features [93]. The majority manifest in new-borns, show predominantly neurological manifestations and can lead to sudden premature death [94]. By investigating patients recruited under this disease category from the 100KGP and looking at human orthologues of EL genes in the mouse for evidence of enrichment of biallelic LoF or predicted pathogenic missense variants, we were able to identify a set of candidate genes where the heterozygous knockout mouse mimicked some neurological and/or metabolic phenotypes observed in patients.

Two of the genes identified through our analysis, *COQ3* and *CDK12*, belong to pathways and extended gene families associated with similar disorders, which strongly supports their involvement in the disease process. Further functional characterisation of these and other predicted pathogenic variants, together with the identification of additional probands with biallelic variants segregating with similar phenotypes, is still needed to establish a causal link, and to confirm that the candidate LoF variants result in the lack of protein product and/or have a discernible clinical phenotypic effect.

The approach described here is based on the premise that biallelic LoF in a gene leads to early embryonic lethality in mice but that biallelic LoF or missense variants in humans lead to recessively inherited metabolic disorders with related phenotypes in humans. In fact, for the four highlighted candidate genes identified in the GEL cohort, it is the heterozygous mouse model which is mimicking the phenotypes observed in patients carrying biallelic mutations. This somehow counterintuitive observation has been reported for other IEM disorders [95, 96]. Most metabolic disorders represent a spectrum of phenotypes. According to OMIM clinical records, more than a third of BIEM genes are associated with lethality before or soon after birth, indicating that a considerable proportion of these conditions in humans are life threatening, leading to early death if untreated. And this proportion is likely an underestimation, given the limited sources of genes linked to prenatal and neonatal lethality in humans. Consistent with this observation, several genes in the same pathway or gene family of our candidate genes (*COQ2*, *COQ4*, *COQ9*, *PDSS2*) [97–100] have been associated with early lethality in humans.

Comparing lethality outcomes between mouse and human presents several limitations. Monoallelic mutations required for early development (dominant lethals) are missing from our set of mouse embryonic lethal knockouts since they would not result in lines, introducing a bias towards recessive lethal genes. Similarly, while in the mouse knockouts the observed phenotype is most likely due to the loss of protein function, other types of mutation may lead to different molecular mechanisms and thus different phenotypic outcomes. True loss of protein function in these genes may be early embryonic lethal in humans whereas postnatal phenotypes could be caused by hypomorphic variants leading to partial LoF [101, 102]. Other explanations include potential mechanisms of compensation through other genes in the pathway in humans or differences in essentiality between the two species. Given the number of genes associated with lethality in the mouse (35% of the knockout lines are classified as lethal or subviable according to IMPC primary viability screening) [24, 25], monogenic factors could explain a proportion of the high and often understated level of occurrence of miscarriages in human [72, 103]. This, together with the potential lack of molecular diagnosis for confirmed miscarriages, leads to an underestimation in current disease databases of embryonic lethality as a Mendelian phenotype [104]. Even when gene essentiality does not perfectly correlate between the two species, the set of lethal genes in the mouse provides knowledge on the molecular functions and biological processes [105] and constitutes an invaluable resource to identify relevant genes in humans, including those for which LoF variation may lead to pregnancy loss and other severe phenotypes with an early manifestation [47].

In summary, the embryonic stage at which lethality occurs in the mouse can be used to inform human disease. Several intolerance to variation scores inferred from human population sequencing data and a broad set of gene features estimate the predicted probability of a gene underlying AR conditions. Our target was a particular subgroup of those genes, associated with BIEM disorders, and in this context our approach outperformed other potential strategies based on existing metrics (Additional file 1: Tables S6-S7). Integration of multi-species datasets and the extended use of standardised phenotypes is key to building novel Mendelian gene discovery approaches [3, 106]. This, coupled with the availability of data from large-scale sequencing programmes that allow for bespoke computational and statistical analysis for variant prioritisation, constitutes a powerful instrument for increasing the molecular diagnostic rate [17]. Additionally, the set of genes essential for embryonic development in the mouse may constitute an additional source of evidence for diagnosis of lethal foetal disorders [47, 107, 108]. Whether this is the only observable outcome or the most extreme phenotype within a wider range of clinical features observed in patients, it will be crucial to catalogue these genes. Several efforts are being made in this area. The foetal medicine community and ontologists are currently working to extend the HPO to cover the prenatal phenotypic manifestations of disease, and including data on the time course of these manifestations, including death will allow further comparisons between mouse and human phenotypes and discrimination between prenatal and postnatal phenotypes [109]. Additionally, we are collating all the information available from OMIM clinical records [6] and the literature to catalogue Mendelian disease genes into lethality categories.

## Conclusions

We have shown cross-species data integration and gene similarity approaches can complement other strategies to identify novel genes underlying Mendelian conditions. In particular, information on knockout mouse embryo lethality can be used to prioritise candidate genes associated with particular types of disorders. Access to unsolved cases from rare disease genome sequencing programmes allows the screening of those genes for potentially pathogenic variants that will hopefully lead to a diagnosis and potentially new treatment options.

## Supplementary Information

**Additional file 1: Table S1.** Gene features: Human cellular essential genes. **Table S2.** Gene features: Gene expression in human brain. **Table S3.** Gene features: Intolerance to variation metrics and paralogues. **Table S4.** Disease features. **Table S5.** HPO phenotypes Odds Ratios. **Table S6.** Comparison of our approach based on EL genes with other strategies based on standard scores thresholds: F-score. **Table S7.** Odds Ratios and 95% CI from multiple logistic regression analysis.

**Additional file 2: Fig. S1.** WoL and cell essentiality scores. **Fig. S2.** WoL and cell essentiality categorisation. **Fig. S3.** WoL and additional gene features. **Fig. S4.** WoL and paralogues features. **Fig. S5.** WoL and additional disease features. **Fig. S6.** Prediction of early lethal genes. **Fig. S7.** Enrichment analysis of genes sharing attributes with a BIEM gene among the EL category.

## Data Availability

All the results presented in the manuscript are available in Supplementary Information (Additional files 1 and 2). All the data supporting the findings of this study are made publicly available in the following repository: 10.5281/zenodo.5796621 [33]. Full viability reports and additional files containing mouse embryo and adult phenotype associations are available through the IMPC web portal (https://www.mousephenotype.org/). Data can be accessed directly through the search box in the homepage, through the batch query tool, via API or via FTP repository (http://ftp.ebi.ac.uk/pub/databases/impc/). More detailed information on how to access and use data and images can be found here: https://www.mousephenotype.org/understand/accessing-the-data/.
