# Supplementary material for: Mendelian gene identification through mouse embryo viability screening
Source: Genome Med. 2022 Oct 13;14:119. doi: 10.1186/s13073-022-01118-7 (PMC9563108; doi:10.1186/s13073-022-01118-7)
Supplement: Supplementary file 1 — Additional file 1: Table S1. Gene features: Human cellular essential genes. Table S2. Gene features: Gene expression in human brain. Table S3. Gene features: Intolerance to variation metrics and paralogues. Table S4. Disease features. Table S5. HPO phenotypes Odds Ratios. Table S6. Comparison of our approach based on EL genes with other strategies based on standard scores thresholds: F-score. Table S7. Odds Ratios and 95% CI from multiple logistic regression analysis. [file 13073_2022_1118_MOESM1_ESM.pdf]

Supplementary Information

Mendelian gene identification through mouse embryo viability screening

Cacheiro et al.

Additional file 1

Content: Supplementary Tables. Tables S1-S7

Table S1. Gene features: Human cellular essential genes. Test for differences in the distribution of CERES scores and proportion of cellular essential genes between WoL. Uncorrected P values for comparison between the three WoL and post hoc pairwise comparisons are shown (see Methods). Alpha threshold for significance was set at 0.0001 given the number of hypothesis tested. WoL, windows of lethality; EL, early gestation lethal; ML, mid gestation lethal; LL, late gestation lethal.

Feature	Figure	EL-ML-LL	EL-ML	ML-LL	EL-LL
Central nervous system	S1a	< 2.2e-50	< 2.2e-50	< 2.2e-50	< 2.2e-50
Blood	S1b	< 2.2e-50	< 2.2e-50	< 2.2e-50	< 2.2e-50
Ovary	S1c	< 2.2e-50	< 2.2e-50	< 2.2e-50	< 2.2e-50
Bile duct	-	< 2.2e-50	< 2.2e-50	< 2.2e-50	< 2.2e-50
Bone	-	< 2.2e-50	< 2.2e-50	< 2.2e-50	< 2.2e-50
Breast	-	< 2.2e-50	< 2.2e-50	< 2.2e-50	< 2.2e-50
Cervix	-	< 2.2e-50	< 2.2e-50	2.1e-26	< 2.2e-50
Colorrectal	-	< 2.2e-50	< 2.2e-50	< 2.2e-50	< 2.2e-50
Embryo	-	< 2.2e-50	1.4e-20	1.2e-03	< 2.2e-50
Engineered	-	< 2.2e-50	< 2.2e-50	1.5e-10	< 2.2e-50
Engineered kidney	-	< 2.2e-50	2.8e-30	2.8e-03	< 2.2e-50
Epidermoid carcinoma	-	< 2.2e-50	8.1e-28	5.1e-03	< 2.2e-50
Esopahagus	-	< 2.2e-50	< 2.2e-50	1.2e-42	< 2.2e-50
Eye	-	< 2.2e-50	< 2.2e-50	5.5e-14	< 2.2e-50
Fibroblast	-	< 2.2e-50	1.1e-28	2.2e-03	< 2.2e-50
Gastric	-	< 2.2e-50	< 2.2e-50	< 2.2e-50	< 2.2e-50
Kidney	-	< 2.2e-50	< 2.2e-50	< 2.2e-50	< 2.2e-50
Liver	-	< 2.2e-50	< 2.2e-50	6.0e-43	< 2.2e-50
Lung	-	< 2.2e-50	< 2.2e-50	< 2.2e-50	< 2.2e-50
Lymphocyte	-	< 2.2e-50	< 2.2e-50	< 2.2e-50	< 2.2e-50
Pancreas	-	< 2.2e-50	< 2.2e-50	< 2.2e-50	< 2.2e-50
Peripheral nervous system	-	< 2.2e-50	< 2.2e-50	3.6e-38	< 2.2e-50
Plasma cell	-	< 2.2e-50	< 2.2e-50	3.3e-48	< 2.2e-50
Prostate	-	< 2.2e-50	< 2.2e-50	8.7e-12	< 2.2e-50
Skin	-	< 2.2e-50	< 2.2e-50	< 2.2e-50	< 2.2e-50
Soft tissue	-	< 2.2e-50	< 2.2e-50	< 2.2e-50	< 2.2e-50
Thyroid	-	< 2.2e-50	< 2.2e-50	2.3e-25	< 2.2e-50
Upper aerodigestive	-	< 2.2e-50	< 2.2e-50	< 2.2e-50	< 2.2e-50
Urinary tract	-	< 2.2e-50	< 2.2e-50	< 2.2e-50	< 2.2e-50
Uterus	-	< 2.2e-50	< 2.2e-50	< 2.2e-50	< 2.2e-50
Cellular essential	2b	< 2.2e-50	1.33e-25	7.42e-09	< 2.2e-50

Table S2. Gene features: Gene expression in human brain. Test for differences in the distribution of gene expression in the brain across different developmental stages between WoL. Uncorrected P values for comparisons between the three WoL and post hoc pairwise comparisons are shown (see Methods). Alpha threshold for significance was set at 0.0005 given the number of hypothesis tested.

Feature	Figure	EL-ML-LL	EL-ML	ML-LL	EL-LL
Brain 4wpc	S3a	8.04e-17	7.03e-05	7.27-03	1.55e-17
Brain 5wpc	S3a	1.45e-17	1.12e-05	1.68e-02	4.81e-18
Brain 7wpc	S3a	8.38e-12	2.31e-05	2.47e-01	8.34e-12
Brain 8wpc	S3a	4.27e-10	2.73e-05	4.94e-01	7.22e-10
Brain 9wpc	S3a	3.39e-15	1.05e-06	2.04e-01	6.54e-15
Brain 10wpc	S3a	4.40e-12	1.41e-05	2.69e-01	5.28e-12
Brain 11wpc	S3a	2.17e-12	1.72e-05	2.14e-01	2.11e-12
Brain 12wpc	S3a	6.87e-08	1.53e-03	2.25e-01	2.53e-08
Brain 13wpc	S3a	5.00e-07	1.39e-03	3.86e-01	2.58e-07
Brain 16wpc	S3a	2.90e-07	8.31e-04	4.37e-01	1.84e-07
Brain 18wpc	S3a	8.40e-07	3.52e-04	7.70e-01	1.14e-06
Brain 19wpc	S3a	3.74e-07	8.76e-04	4.51e-01	2.40e-07
Brain 20wpc	S3a	1.71e-07	1.10e-03	3.37e-01	8.53e-08
Brain newborn	S3a	3.35e-04	1.31e-02	6.08e-01	1.54e-04
Brain infant	S3a	1.04e-06	7.18e-03	1.78e-01	2.68e-07
Brain toddler	S3a	1.95e-05	1.10e-02	3.10e-01	6.06e-06
Brain school	S3a	4.61e-07	2.75e-03	2.61e-01	1.64e-07
Brain teenager	S3a	6.85e-07	5.46e-03	1.90e-01	1.87e-07
Brain youngAdult	S3a	3.57e-05	1.79e-02	2.69e-01	9.67e-06
Brain youngMidAge	S3a	3.43e-05	2.23e-02	2.28e-01	8.46e-06
Brain olderMidAge	S3a	2.80e-06	8.92e-03	2.04e-01	7.38e-07
Brain senior	S3a	5.67e-07	6.47e-03	1.60e-01	1.41e-07

Table S3. Gene features: Intolerance to variation metrics and paralogues. Test for differences in the distribution of several intolerance to variation metrics between WoL. Uncorrected P values for comparisons between the three WoL and post hoc pairwise comparisons are shown (see Methods). Alpha threshold for significance was set at 0.001 given the number of hypothesis tested. WoL, windows of lethality; EL, early gestation lethal; ML, mid gestation lethal; LL, late gestation lethal; MoI, mode of inheritance.

Feature	Figure	EL-ML-LL	EL-ML	ML-LL	EL-LL
SCoNeS	2d	1.56e-06	3.96e-03	2.91e-01	5.45e-07
LOEUF	2e	3.71e-04	6.70e-03	8.59e-01	2.69e-04
RVIS	S3c	4.51e-01	2.88e-01	2.19e-01	7.72e-01
pLI	S3d	1.06e-02	7.20e-03	3.46e-01	2.87e-02
pRec	S3e	4.71e-02	3.78e-02	5.98e-01	5.01e-02
DOMINO	S3f	1.89e-05	1.66e-02	2.31e-01	4.83e-06
DOMINO Very likely recessive	S3g	9.45e-05	2.94e-02	3.77e-01	3.39e-05

Singletons	2f	1.41e-20	1.13e-04	2.57e-04	4.87e-21
Opisthokonta-Bilateria	S4a	2.37e-08	1.78e-03	1.91e-01	7.32e-09
Number of paralogues	S4b	9.40e-05	5.11e-03	6.56e-01	3.14e-05
WoL of paralogues EL	S4c	3.22e-06	1.45e-02	1.89e-01	1.47e-06
Singletons – cellular essential EL	S4d	4.65e-01	-	-	-
Singletons – cellular essential ML	S4d	2.52e-03	-	-	-
Singletons – cellular essential LL	S4d	1.87e-01	-	-	-

Table S4. Disease features. Test for differences among WoL. Uncorrected P values for comparisons between the three WoL and post hoc pairwise comparisons are shown (see Methods). The number of observations was too small in some groups given the low number of disease associated genes for certain categories. Alpha threshold for significance was set at 0.001 given the number of hypothesis tested. WoL, windows of lethality; EL, early gestation lethal; ML, mid gestation lethal; LL, late gestation lethal; MoI, mode of inheritance.

Feature	Figure	EL-ML-LL	EL-ML	ML-LL	EL-LL
Mendelian genes	3a	3.07e-05	3.31e-02	2.23e-01	9.08e-06
MoI biallelic	3b	1.32e-05	7.65e-03	4.17e-01	5.16e-06
Neurology	3c	5.31e-01	1.000	4.96e-01	3.68e-01
Metabolic	3c	2.70e-06	2.52e-02	1.52e-01	1.05e-06
Skeletal	3c	4.57e-02	2.58e-02	4.35e-01	1.15e-01
Ophthalmological	S5a	-	-	-	-
Dysmorphic	S5a	5.09e-01	6.00e-01	1.000	3.40e-01
Endocrine	S5a	2.09e-01	3.72e-01	1.000	1.32e-01
Cardiovascular	S5a	-	-	-	-
Ciliopathies	S5a	-	-	-	-
Dermatological	S5a	-	-	-	-
Gastroenterological	S5a	-	-	-	-
Growth	S5a	-	-	-	-
Haematological	S5a	-	-	-	-
Hearing	S5a	-	-	-	-
Renal	S5a	3.35e-01	5.53e-01	9.44e-01	2.07e-01
Respiratory	S5a	-	-	-	-
Rheumatological	S5a	-	-	-	-

Table S5. HPO phenotypes Odds Ratios. Odds Ratio with 95% CI and BH adjusted P values for EL IEM genes compared to NEL (mid gestation lethal + late gestation lethal) IEM genes. No significant differences were found for any of the top level HPO phenotypes, corresponding to physiological systems. EL, early gestation lethal; HPO, human phenotype ontology; IEM, inborn errors of the metabolism; CI, confidence intervals; BH, Benjamini-Hochberg.

Feature	OR	OR lower	OR upper	P-value	Adjusted P-value
Abnormality of the nervous system	1.36	0.29	6.36	0.722	0.844
Abnormality of head or neck	1.69	0.75	3.86	0.22	0.524
Abnormality of metabolism/homeostasis	0.37	0.08	1.34	0.146	0.524
Growth abnormality	1.98	0.83	4.81	0.128	0.524
Abnormality of the integument	1.66	0.76	3.68	0.238	0.524
Abnormality of the genitourinary system	0.54	0.24	1.20	0.162	0.524
Abnormality of the respiratory system	0.78	0.36	1.71	0.559	0.821
Abnormality of limbs	0.94	0.43	2.05	1.000	1.000
Abnormality of the digestive system	0.79	0.31	1.95	0.653	0.844
Abnormality of the endocrine system	0.86	0.37	2.00	0.832	0.872
Abnormality of the cardiovascular system	1.21	0.53	2.74	0.683	0.844
Abnormality of blood and blood-forming tissues	1.38	0.62	3.11	0.546	0.821
Abnormality of the immune system	0.59	0.26	1.33	0.22	0.524
Abnormality of the musculoskeletal system	0.66	0.13	2.75	0.729	0.844
Abnormality of prenatal development or birth	0.69	0.27	1.75	0.485	0.821

Table S6. Comparison of our approach based on EL genes with other strategies based on standard scores thresholds: F-score. Precision and Recall were computed based on the number of predicted recessive genes using the suggested thresholds for the different scores (Genes in category), and the number of BIEM genes in each of these sets of candidate genes. Only those genes with data available for all the metrics were considered for the analysis. BIEM, biallelic inborn errors of the metabolism; EL, early gestation lethal.

	EL	pRec > 0.90	DOMINO very likely recessive	SCoNeS > 0.75	LOEUF < 0.35
BIEM genes (with data available for all the metrics)	223	223	223	223	223
Genes in category (with data available for all the metrics)	744	1589	2889	3357	993
BIEM genes & Genes in category	76	105	172	204	5
F-score	0.156	0.116	0.111	0.114	0.008

Table S7. Odds Ratios and 95% CI from multiple logistic regression analysis. A logistic regression model was fitted using the different scores (continuous) and EL (binary, EL vs ANEL) as predictors. The response binary variable indicates whether a gene belongs to the BIEM class or not. OR were computed for specific increment step of continuous variable. EL, early gestation lethal; ANEL, all non-early gestation that includes mid and late gestation lethal, subviable and viable genes; BIEM, biallelic inborn errors of the metabolism; OR, Odds Ratio; CI, confidence interval

Predictor	OR	CI Low (2.5)	CI High (97.5.5)	Increment
WoL EL	5.014	3.636	6.884	indicator variable
DOMINO	1.087	0.960	1.224	0.1
SCoNeS	2.593	2.084	3.273	0.1
LOEUF	0.963	0.918	1.010	0.1
pRec	0.954	0.908	1.003	0.1
