# Supplementary material for: Mendelian gene identification through mouse embryo viability screening
Source: Genome Med. 2022 Oct 13;14:119. doi: 10.1186/s13073-022-01118-7 (PMC9563108; doi:10.1186/s13073-022-01118-7)
Supplement: Supplementary file 2 — Additional file 2: Fig. S1. WoL and cell essentiality scores. Fig. S2. WoL and cell essentiality categorisation. Fig. S3. WoL and additional gene features. Fig. S4. WoL and paralogues features. Fig. S5. WoL and additional disease features. Fig. S6. Prediction of early lethal genes. Fig. S7. Enrichment analysis of genes sharing attributes with a BIEM gene among the EL category. [file 13073_2022_1118_MOESM2_ESM.pdf]

Supplementary Information

Mendelian gene identification through mouse embryo viability screening

Cacheiro et al.

Additional file 2

Content: Supplementary Figures. Figures S1-S7

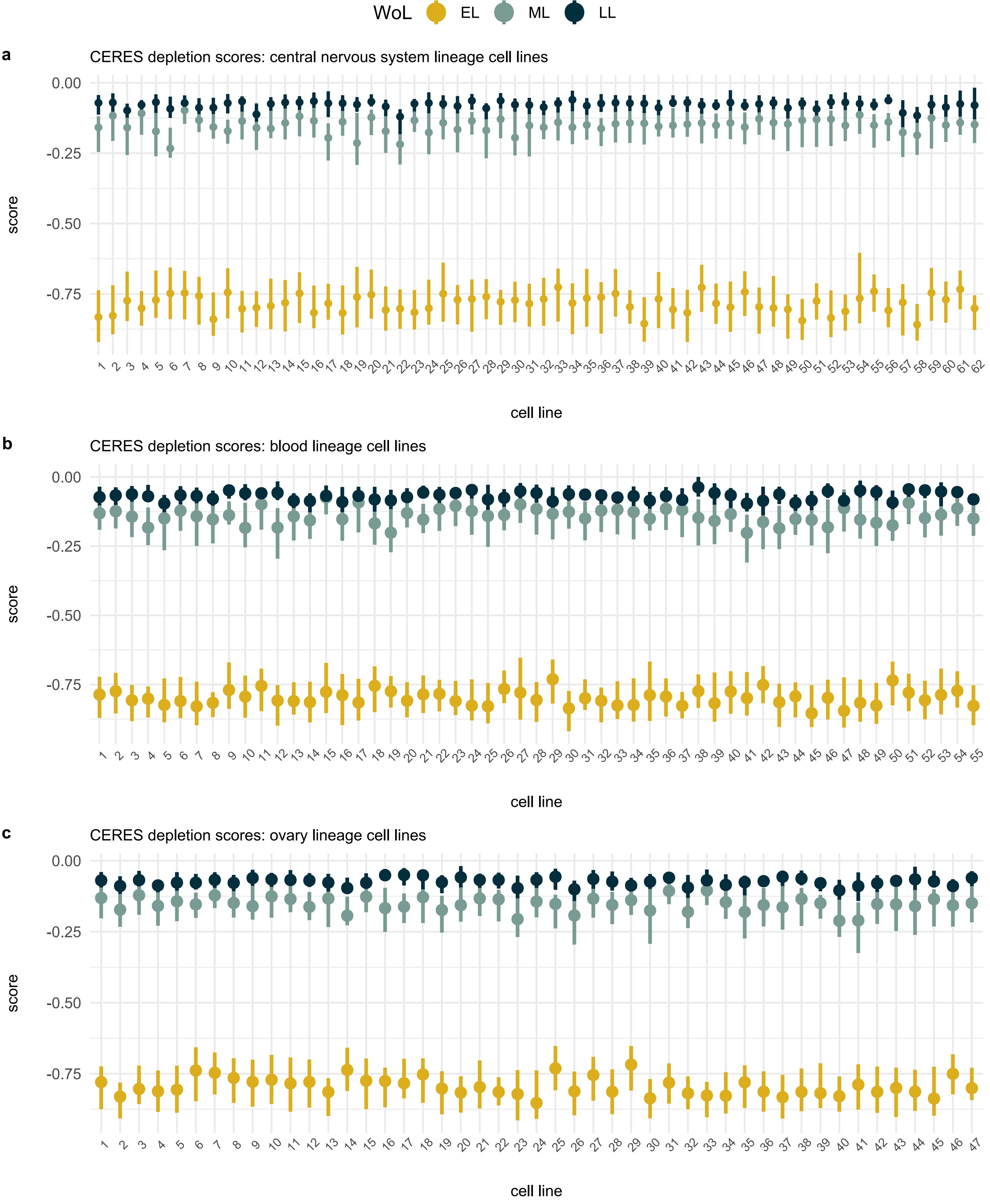

Fig. S1. WoL and cell essentiality scores. a CERES depletion scores for different central nervous system human cell lines. Median scores and the corresponding lower and upper bounds of the 95% CI for different central nervous system cell lines and each WoL. **b CERES depletion scores for blood lineage human cell lines.** Median scores and the corresponding lower and upper bounds of the 95% CI for different blood cell lines and each WoL. **c CERES depletion scores for ovary lineage human cell lines**. Median scores and the corresponding lower and upper bounds of the 95% CI for different ovary cell lines and each WoL. A more negative score indicates more depletion of the gene in the cell line, i.e. more essential. The results for the statistical tests of significance are available in Table S1. WoL, windows of lethality; EL, early gestation lethal; ML, mid gestation lethal; LL, late gestation lethal; CI, confidence interval

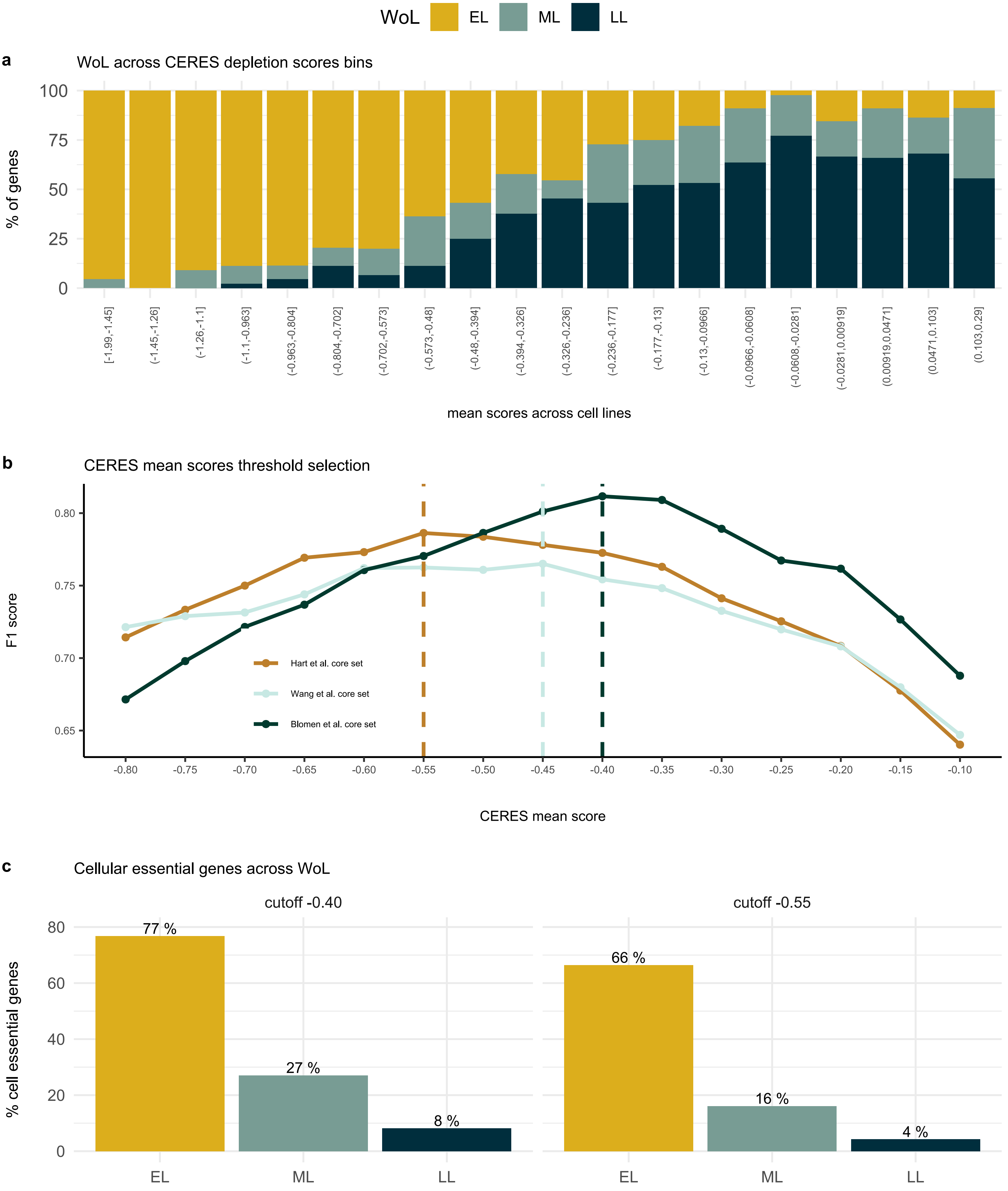

Fig. S2. WoL and cell essentiality categorisation. a WoL across mean CERES depletion scores. Barplots show the proportion of genes in each WoL for mean CERES score bins of equal size. **b Binary categorisation of CERES depletion scores.** Genes are categorised as essential and non-essential based on a threshold to maximize F1 score using a binary classification from previous datasets. **c WoL and cellular essential genes**. Percentage of EL, ML and LL genes considered cellular essential when alternative thresholds (-0.40 and -0.55) are considered. WoL,windows of lethality; EL, early gestation lethal; ML, mid gestation lethal; LL, late gestation lethal; CI, confidence interval.

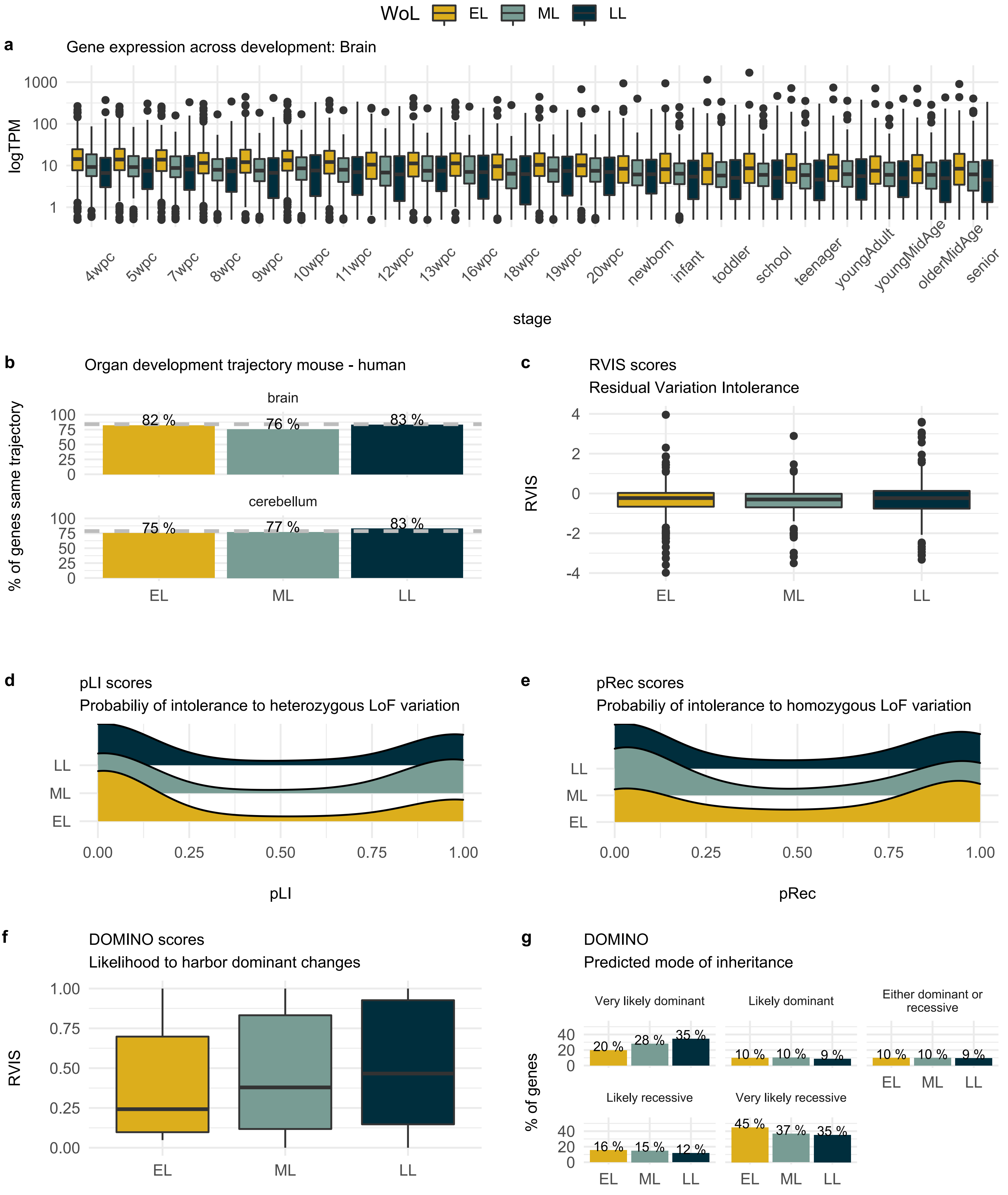

Fig. S3. WoL and additional gene features. a Gene expression in brain. Boxplots show the distribution of gene expression values for genes within each WoL across developmental stages for human brain. **b WoL and organ development trajectories**. Percentage of genes in each WoL with the same organ development trajectory between mouse and human (brain and cerebellum). They grey dashed line shows the baseline %, corresponding to the percentage of genes showing no significantly different trajectories for the entire set of genes with data available. **c WoL and RVIS scores**. Boxplots show the distribution of RVIS scores, which measure the tolerance of a gene to contain damaging variation, across WoL. **d WoL and pLI scores**. Distribution of pLI scores for each WoL, showing the bimodal distribution of this score. **e WoL and pRec scores**. Distribution of pRec scores for each WoL, showing the bimodal distribution of this score. **f WoL and DOMINO scores**. Boxplots show the distribution of DOMINO scores that assess the likelihood for a gene to harbour dominant change across WoL.

g WoL and DOMINO categories. Barplots show the percentage of genes belonging to each one of the predicted mode of inheritance categories according to DOMINO scores for the three WoL. The results for the statistical tests of significance are available in Tables S2-S3. WoL, windows of lethality; EL, early gestation lethal; ML, mid gestation lethal; LL, late gestation lethal; pLI, probability of being intolerant to heterozygous LoF variation; pRec, probability of being intolerant to homozygous LoF variation.

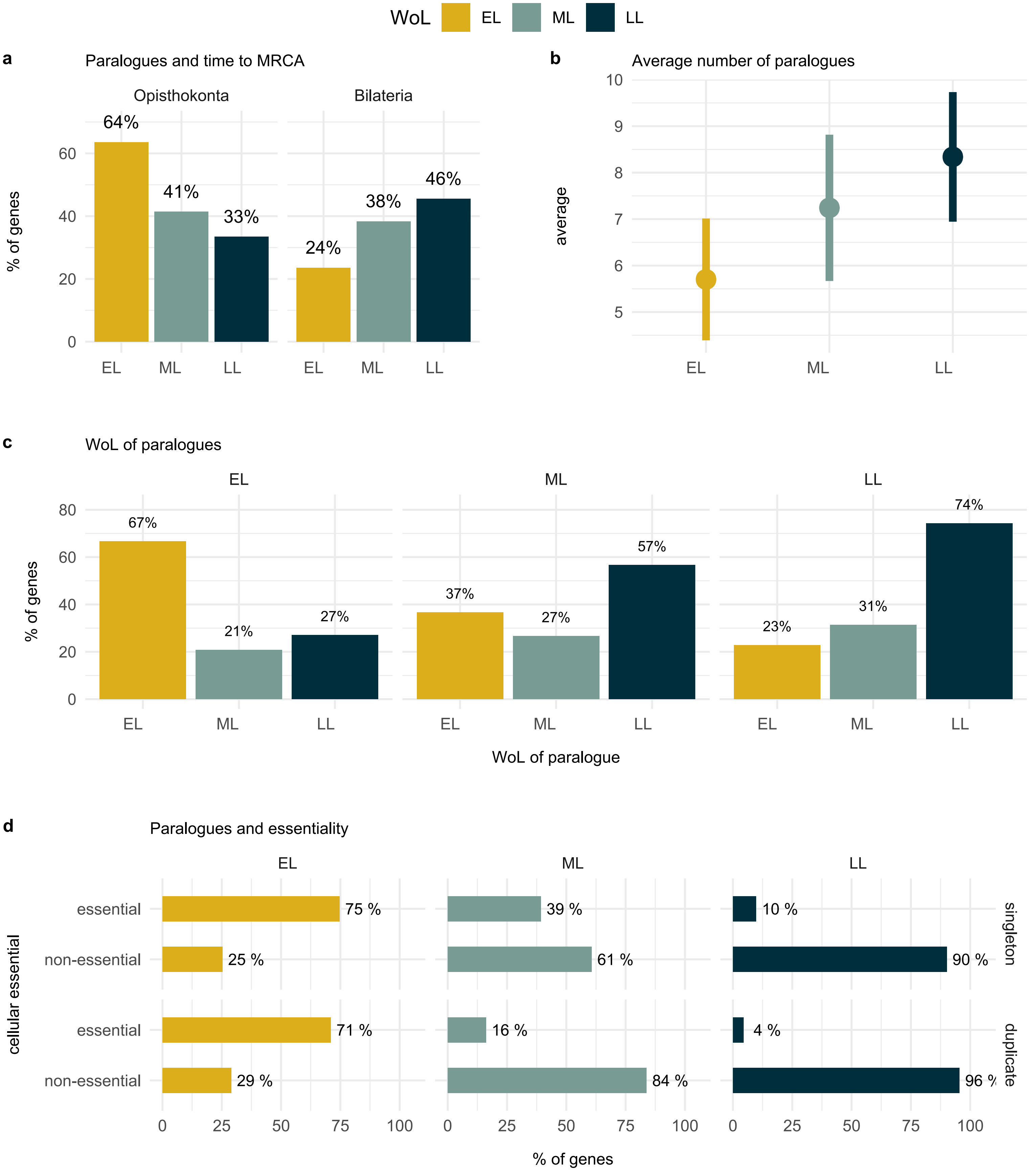

Fig. S4. WoL and paralogues features. a Paralogues and time of the duplication event. The time of the duplication event which produced the paralogue is assessed by identifying the most recent common ancestor (MRCA). The taxons Opisthokonta (older) and Bilateria (more recent) represent the most frequent ones. The barplots represent the percentage of paralogues for each WoL and time of the duplication event. **b Average number of paralogues**. Average and 95% CI of the number of paralogues per gene for those genes with duplicates across WoL. **c WoL of paralogues**. Distribution of the WoL of the paralogue genes for each WoL. For each WoL, the percentage of paralogue genes mapping each WoL is shown. **d WoL, paralogues and cellular essentiality**. For each WoL, genes are divided into singletons (no paralogues) and duplicates (paralogues), and the barplots represent the percentage of cellular essential and non-essential genes. The results for the statistical tests of significance are available in Table S3 . WoL, windows of lethality; EL, early gestation lethal; ML, mid gestation lethal; LL, late gestation lethal; CI, confidence interval.

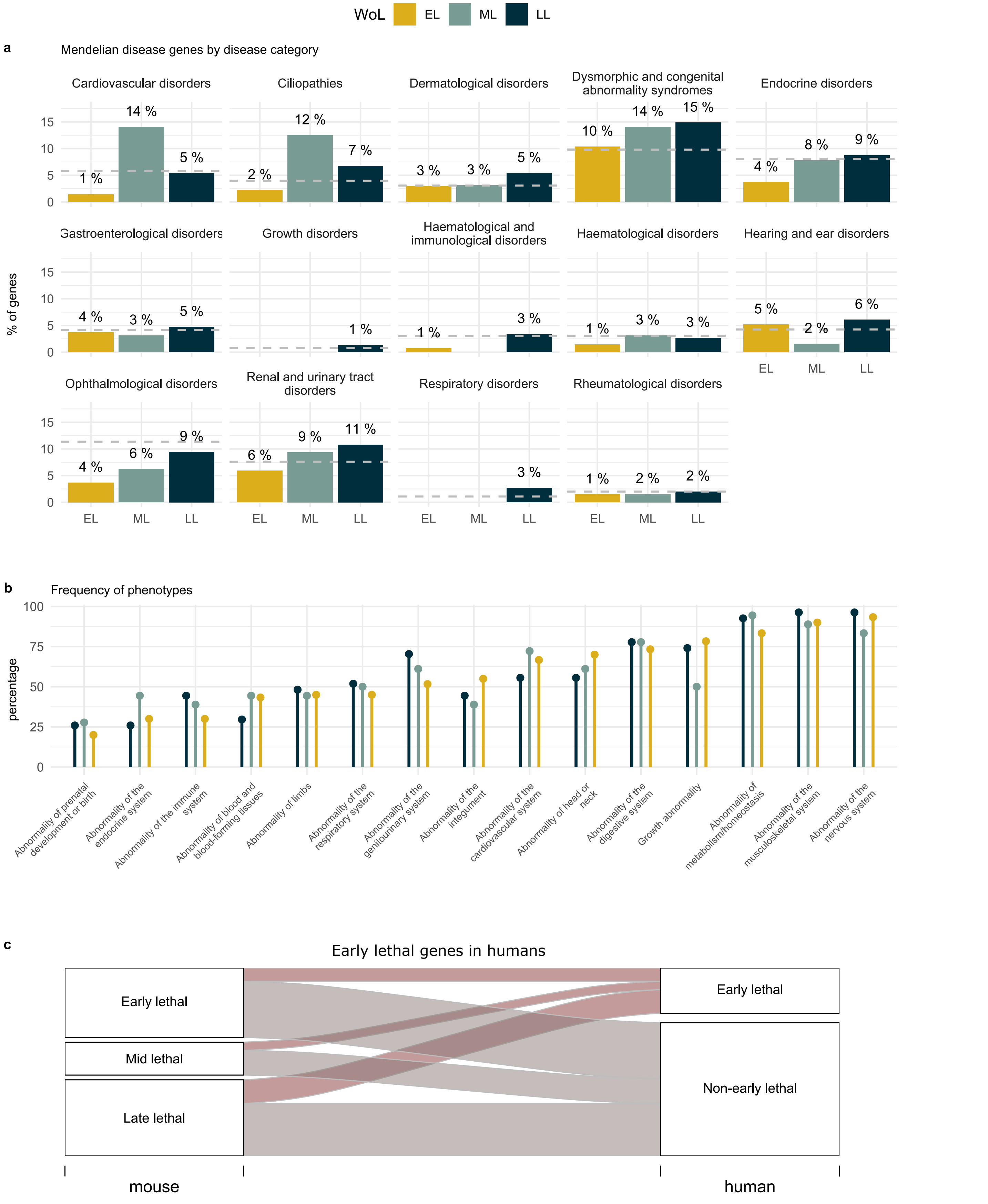

Fig. S5. WoL and additional disease features. a Mendelian disease genes. Barplots showing the frequency of disease associated genes for the remaining disease categories across WoL. The grey dashed line shows the baseline %, corresponding to the percentage of genes in each disease category with respect to the total number of PanelApp "green" genes (3,384). **b Top level HPO annotations**. Frequency of top-level HPO phenotype annotations for inborn errors of metabolism genes in each window. **c WoL and early lethality in humans**. Human early lethal genes: PanelApp 'green' genes associated with early lethality (see Methods); human non early lethal genes: the remaining set of PanelApp 'green' genes. Tests for differences between WoL are available in Table S4. WoL, windows of lethality; EL, early gestation lethal; ML, mid gestation lethal; LL, late gestation lethal; HPO, human phenotype ontology.

a

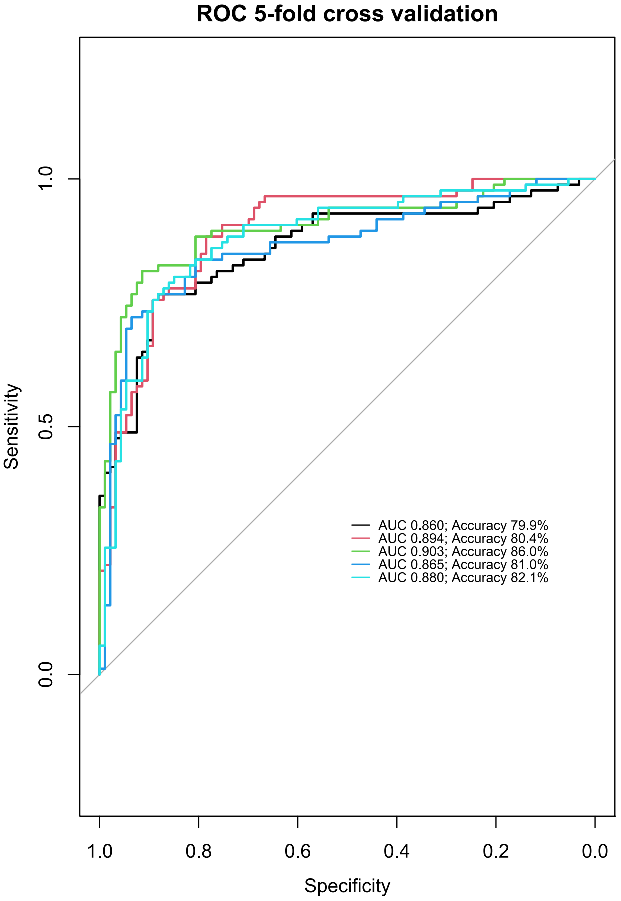

b

	DepMap_ID	lineage
1	ACH-000045	blood
2	ACH-000141	bile_duct
3	ACH-000257	lung
4	ACH-000272	kidney
5	ACH-000313	kidney
6	ACH-000318	esophagus
7	ACH-000459	kidney
8	ACH-000537	liver
9	ACH-000573	breast
10	ACH-000618	upper_aerodigestive
11	ACH-000677	lung
12	ACH-000760	central_nervous_system
13	ACH-000851	lung
14	ACH-000969	colorectal
15	ACH-001041	lung
16	ACH-001061	colorectal
17	ACH-001163	kidney
18	ACH-001232	central_nervous_system
19	ACH-001307	thyroid
20	ACH-001518	uterus
21	ACH-001628	ovary
22	ACH-001688	kidney
23	ACH-001737	blood
24	ACH-001786	colorectal

Fig. S6. Prediction of early lethal genes. A penalised likelihood approach was used to fit a generalised additive model using proliferation (essentiality) scores from multiple cell lines as predictors and subsequently used that model to make the predictions. The effect of each variable is estimated to be either zero, linear, or a low-complexity curve. **a ROC-AUC**. 5-fold CV ROC-AUC and accuracy estimated on a balanced set of 895 genes (430 EL, 465 NEL). ROC-AUC values ranged from 0.860 to 0.903. The accuracy ranged from 79.9% to 86.0% of instances correctly classified as EL and NEL. **b Predictor variables**. Human cancer cell lines with DepMap CERES scores with non-zero coefficients. Only 24 cell lines out of 902 were selected in the final model. CV, Cross validation; ROC-AUC, Area Under the Receiver Operating Characteristic Curve; EL, early gestation lethal; NEL, non-early gestation lethal (mid gestation lethal and late gestation lethal).

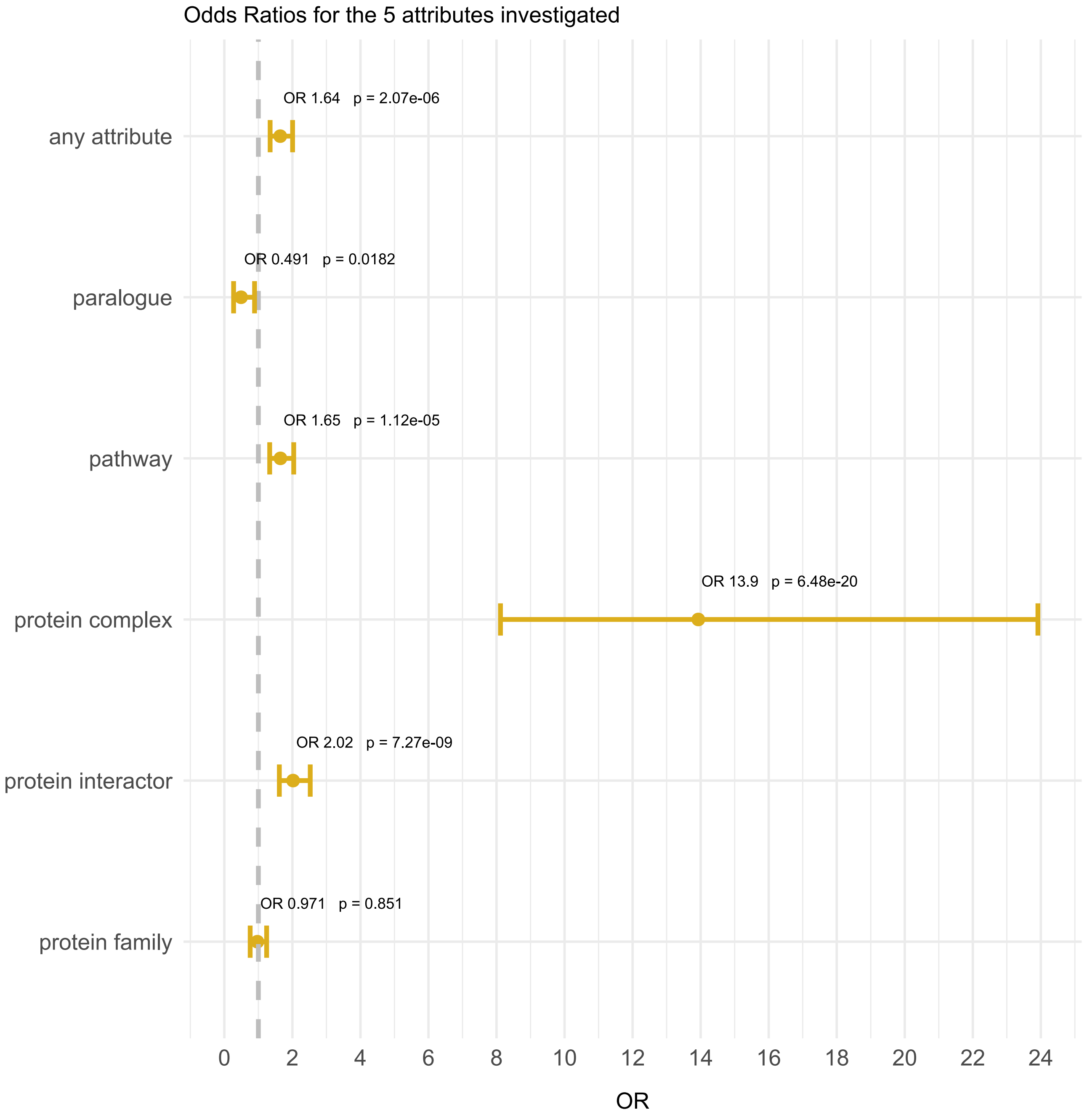

Fig. S7. Enrichment analysis of genes sharing attributes with a BIEM gene among the EL category. Odds Ratios with 95% confidence intervals and BH adjusted P values for the five attributes investigated. EL genes were compared to ANEL genes (mid gestation lethal, late gestation lethal, subviable and viable genes). EL, early lethal genes; ANEL, all non-early gestation lethal genes; BH, Benjamini-Hochberg.
